# *Pseudomonas alliivorans, Pseudomonas asturiensis*, and *Pseudomonas viridiflava* multi-infections and competitive interactions in wilted plants of *Rosmarinus officinalis* L

**DOI:** 10.3389/fpls.2026.1799303

**Published:** 2026-04-15

**Authors:** Giulia Semenzato, Eliana Dell’Olmo, Aida Raio, Giovanna Serratore, Sihem Fodil, Giovanni Ragosta, Loredana Sigillo

**Affiliations:** 1 National Research Council, Institute for Sustainable Plant Protection, Sesto Fiorentino, Italy; 2 Council for Agricultural Research and Economics—Research Centre for Vegetable and Ornamental Crops, Pontecagnano Faiano, Italy

**Keywords:** co-inoculation, LOPAT, MLSA, pathogen competition, rosemary

## Abstract

*Rosmarinus officinalis* L. is an evergreen shrub widely cultivated as an ornamental plant and for its bioactive compounds. However, the increasing intensification of rosemary cultivation has led to the emergence of phytosanitary problems caused by fungal and bacterial pathogens. In 2018, rosemary plants showing branch knot symptoms and severe desiccation were collected from an open-field cultivation in Southern Italy. Bacterial isolation from symptomatic tissues yielded several colonies with pseudomonad-like features. In this study, an accurate diagnosis of the observed disease was performed, together with the molecular, biochemical, and physiological characterization of the isolates and the assessment of their pathogenicity and interactions. Bacterial identification based on LOPAT tests, BIOLOG GEN III microplates, and multi-locus sequence analysis revealed the presence of *Pseudomonas viridiflava*, *P. asturiensis*, and *P. alliivorans*. All three species were pathogenic to rosemary, although with different levels of virulence, with *P. alliivorans* causing the most severe symptoms. Two- and three-species co-inoculation assays on rosemary and tomato plants indicated that competitive interactions predominated among the strains, with no evidence of synergistic effects on disease development. These results were supported by *in vitro* assays, which revealed antagonistic behaviors and mutual inhibition among the species. Overall, this study provides new insights into the complex interactions among *Pseudomonas* species involved in rosemary disease and highlights the role of interspecific competition in shaping disease outcomes.

## 1 Introduction

*Rosmarinus officinalis* L. is an evergreen shrub belonging to the Lamiaceae family. It originates from the Mediterranean area but is globally cultivated as an ornamental plant and for its leaves, which are rich in active compounds utilized as food aromas, antimicrobials, and for many other medical applications (Datiles and Acevedo-Rodríguez, 2022; Qiu et al., 2024). Essential oils and phenolic extracts, which are responsible for the biological activities and beneficial health properties of rosemary (Ribeiro-Santos et al., 2015), are recognized as safe by the FDA and EFSA (Bellumori et al., 2021). Hence, an economic interest in the rosemary plants as an open field or a protected crop cultivation has increased over the years. However, the intensification of cultivation has led the growers to face serious phytosanitary problems, caused by fungal and bacterial pathogens that are responsible for root and stem damage to plants. *Phytophtora nicotianae* (Álvarez et al., 2007), *Fusarium* spp. (Mekonnen et al., 2019; Dell’Olmo et al., 2025), *Rhizoctonia* spp. (Garibaldi et al., 2013; Azcona et al., 2017), and *Botrytis cinerea* (Pscheidt, 2021) are the most destructive fungal pathogens of rosemary, causing severe economic losses. Regarding the pathogenic bacteria, *Xylella fastidiosa* subsp. *multiplex* has been recently detected on rosemary plants collected from a natural park in Portugal, where a disease outbreak was reported on different hosts (Carvalho-Luis et al., 2022), while *Xanthomonas* sp. and *Pseudomonas viridiflava* are well-known pathogens responsible for leaf spot diseases of rosemary (McGovern, 2023; Martini et al., 2009).

The genus *Pseudomonas* comprises several pathogenic species that are currently classified within species complexes. *Pseudomonas syringae* complex (PSC) includes nine genomospecies, 13 phylogroups, 15 recognized bacterial species, and more than 60 different pathovars of the *sensu stricto* species *Pseudomonas syringae* (Berge et al., 2014; Gardan et al., 1999; Gomila et al., 2017). However, over the years, new species have been identified and ascribed to the PSC simultaneously with the availability of new molecular methodologies. *P. viridiflava* is a wide host range pathogen which may behave as an epiphyte, endophyte, or saprophyte in both agricultural and natural environments (Lipps and Samac, 2022). Even though *P. viridiflava* is grouped within the PSC, this species shows its own peculiar characteristics, including pectate lyase as a virulence factor, atypical pathogenicity islands, and phenotypic variation (Lipps and Samac, 2021). Moreover, in the last decades, several studies reported the isolation, from different host plants, of atypical *P. viridiflava* showing yellow mucoid colonies and/or variable response to LOPAT tests (González et al., 2003; Myung et al., 2010; Cariddi et al., 2024). Three different *Pseudomonas* species were isolated from wilted rosemary plants, evidencing a putative case of a multi-infection disease. These diseases are complex, and it is difficult to evaluate the role of the single pathogens and the outcome of their mutual interaction, including those with the plant. Moreover, few reports are available in the literature. This work aims: i) to carry out an accurate diagnosis of a disease observed in rosemary plants based on pathogenicity test; ii) to describe the bacterial isolates obtained from rosemary plants by molecular, biochemical, and physiological characterization; iii) to investigate the *in vitro* and *in vivo* interactions among the bacterial species involved to highlight their putative role in disease expression; iv) to describe a new disease of rosemary where multiple bacterial agents could be involved.

## 2 Materials and methods

### 2.1 Field disease observation and bacterial isolation

**Figure 1:**
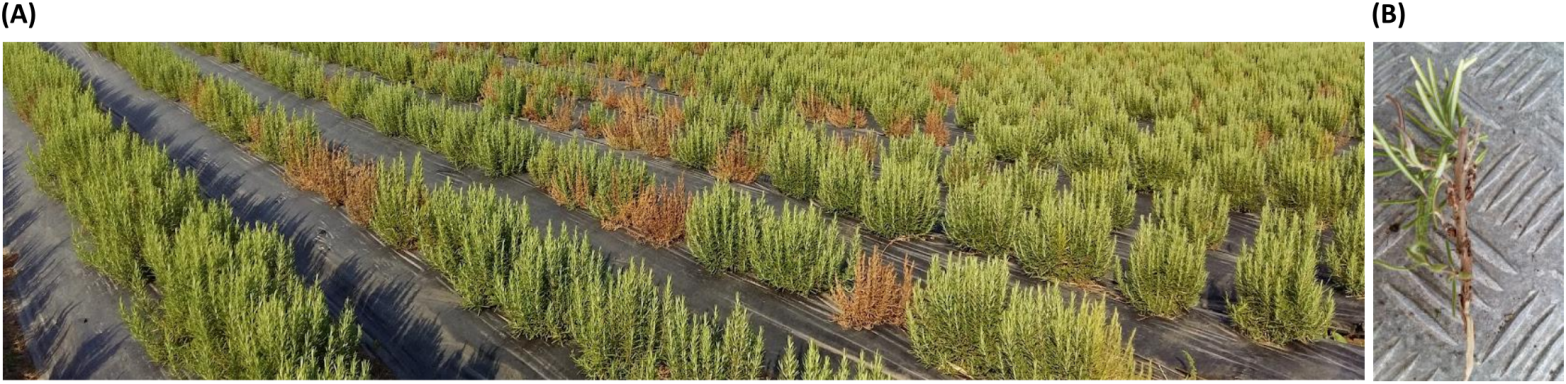
Disease manifestation. **(A)** Rosemary field; **(B)** damage on the plant stem.

In June 2018, in an open-field cultivation (Battipaglia, Italy) of an unknown rosemary variety, heavy wilting symptoms were observed in nearly 10% of the plants. The disease was visible in patches along the rows. The aerial part of the plants appeared wilted with desiccation extended asymmetrically to one or more branches. On stems, along the woody portion, hypertrophies were observed and leaves often fell (**Figure 1**). In plants with an advanced disease status, the roots were necrotized.

10 symptomatic plants were transferred to the “Laboratory of diagnostic and disease resistance testing” of CREA Research Centre for Vegetable and Ornamental crops in Pontecagnano (Italy), for diagnostic analyses. Plant samples were processed for detecting fungal at the root and collar levels and for bacterial pathogens in the knot tissues. To these aims, necrotic roots and collars were surface disinfected with 1% NaClO for 30 minutes and then rinsed three times in sterile distilled water (SDW). They were subsequently dried under a laminar flow cabinet, 20 pieces of 5 mm were cut from each basal plant portion, and incubated at 25 ± 1 °C on Potato Dextrose Agar (PDA, Oxoid, Thermofisher, Foster City, CA, USA) amended with streptomycin 100 ppm, neomycin 50 ppm, and chloramphenicol 50 ppm (all the antibiotics were purchased from Merck, Darmstadt, DE). After 1 week, PDA plates were observed for the development of fungal colonies. Knots were excised from the stems and disinfected as described above, performing a 10-minute NaClO treatment. Stem pieces of 1 cm were dried under a laminar flow cabinet and ground in sterile mortars containing 3 ml of sterile saline solution (SS). The tissue macerates were left standing for 10 minutes to allow the separation of liquid suspension from the coarse plant tissue debris. Suspensions were diluted 10-fold until 10^-7^. 100 µL of each dilution were spread on Nutrient Agar (Oxoid Thermofisher, Foster City, CA, USA) amended with D-glucose 2.5 g/L (NGA), and the plates were incubated at 28 ± 1 °C for one week. Starting from 3 days after plate incubation, colonies representing different morphologies were picked up and streaked onto NGA medium for purification. Pure bacterial colonies were resuspended in double-distilled sterile water and incubated at + 4 °C for medium-term storage, and in 30% glycerol for long-term storage at -80 °C in the MicroHort collection of CREA.

### 2.2 Bacterial strain characterization

All isolates (named 1N2, 1N3, 1N4, 2N5, 2N8, 2N9, 3N1, and 3N3) were assayed by the KOH test to determine the Gram reaction (Buck, 1982). The 8 bacterial colonies were streaked on King B agar medium (Sigma-Aldrich, CH) to verify the presence of diffusible fluorescent pigmentation and then submitted to LOPAT characterization according to Lelliot and Stead (1987). The determinative LOPAT tests, including levan production, oxidase production, pectolytic activity, arginine dihydrolase production, and tobacco hypersensitive reaction (HR), were performed according to the protocols reported by the authors (Lelliot and Stead, 1987). For the pectolytic activity test, to reduce the risk of contamination, potato tubers were surface sterilized with 1% NaClO for 20 minutes and then rinsed three times in SDW. Potato slices 6–7 mm thick were cut with a sterile blade and placed on the bottom of a disposable 9 mm diameter Petri dish, which was then placed inside a sterile glass dish of 12 cm diameter. Wetted sterile filter papers were placed on the bottom of the dish. The glass dishes were closed with their lid, sealed with extensive film, and incubated at 26 ± 1 °C for 2 days. HR was performed at room temperature on the tobacco plants cv. Burley.

### 2.3 DNA extraction, amplification, and sequencing

Strains 1N2, 1N3, 1N4, 2N5, 2N8, 2N9, 3N1, and 3N3 were grown on NGA plates for 48 h at 27 °C, then a few colonies were suspended in SS and suspensions adjusted to OD = 0.1 at 600 nm. One milliliter of each suspension was transferred to sterile microtubes and incubated at 95 °C for 15’, cooled on ice, and centrifuged at 8000 rpm for 5 minutes. The supernatants were transferred to new sterile microtubes. DNA concentration was determined by a spectrophotometer (Tecan Infinite M Plex, Tecan Trading AG, Switzerland). Amplification of 16S rDNA, *gyrB*, *rpoB*, and *rpoD* regions was performed for all 8 isolates using the primers listed in **Supplementary Table 1**, following the protocols described in the respective references (Ait Tayeb et al., 2005, Mulet et al., 2009, Weisburg et al., 1991, Yamamoto et al., 2000). Amplicons (**Supplementary Figure 1**) were purified using the mi-PCR Purification Kit (Metabion International, Germany) and sequenced through Sanger sequencing technology. DNA sequencing was performed by BMR Genomics srl (Padua, Italy). Gene sequences were visualized using Chromas and trimmed at the point where sequence quality was scarce. The BLASTn tool (http://blast.ncbi.nlm.nih.gov/) was then used to identify the most similar sequences in the NCBI nucleotide database, with the “sequences from type material” option enabled. Sequences were submitted to GenBank and assigned the following accession numbers: from PX857324 to PX857331 (16S rRNA gene), from PX896467 to PX896474 (*gyrB*), from PX896475 to PX896482 (*rpoB*), and from PX896483 to PX896490 (*rpoD*).

### 2.4 Phylogenetic tree construction

Based on BLASTn preliminary results, available annotated genomes of *P. viridiflava*, *P. alliivorans*, and *P. asturiensis* were selected as references for a MultiLocus Sequence Analysis (MLSA), while a few *P. syringae*, *P. syringae* pv. *primulae*, and *P. fluorescens* submitted genomes were chosen as outgroups. In particular, the sequences of the 16S rDNA, *gyrB*, *rpoB*, and *rpoD* genes were downloaded from the GenBank Assembly database. Sequences were aligned with those of the isolates obtained in this study and subsequently concatenated. Specifically, 756 bp (16S rDNA), 565 bp (*gyrB*), 474 bp (*rpoB*), and 570 bp (*rpoD*) were used. Concatenated sequences were aligned in MEGA12 using the Muscle algorithm. Unmatched bases at the beginning and at the end of the aligned sequences were manually trimmed. Phylogenetic analyses were conducted in MEGA12, utilizing up to three parallel computing threads. The evolutionary history was inferred using the Neighbor-Joining method with a 1000-bootstrap resampling, while distances were estimated using the Kimura 2-parameter method. The pairwise deletion option was applied.

### 2.5 Pathogenicity tests and disease observation after multiple infections

**Figure 2:**
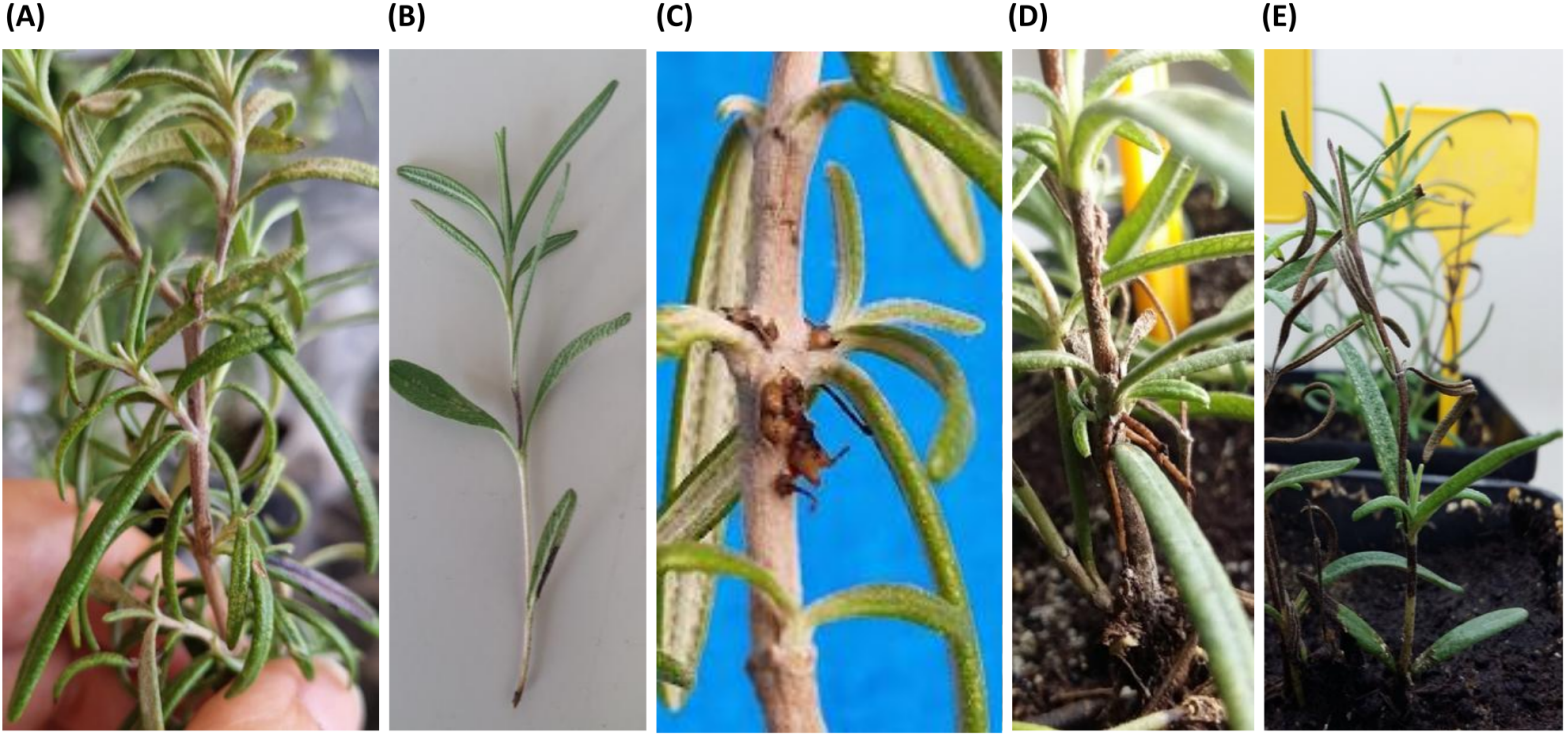
Disease scale used to measure symptom level in rosemary: **(A)** level 0, no symptoms; **(B)** level 1, discoloration at the inoculation point; **(C)** level 2, browning and hypertrophy; **(D)** level 2, presence of adventitious roots; **(E)** level 3, wilted plant.

Pathogenicity of three selected strains was determined by inoculating 6 rosemary cuttings for each strain, obtained from the stems of mother plants. Cuttings were transplanted into 10 cm pots containing peat and grown for 1 month in a climatic chamber at 26 °C. In June, plants were moved to a glasshouse and grown for 3 months at a maximum temperature of 28 °C, with regular irrigation. After 1 month, each strain was inoculated on 3 wounds (made with a sterile needle at the branch insertions), above the lowest branch, in the middle of the stem, and on the herbaceous apical part of each plant. 10 µL of each 10^8^ CFU/mL bacterial suspension were placed on the wounds. Six plants treated with SDW were used as a negative control. Inoculated plants were covered with a plastic sheet for 24 hours and grown in a greenhouse with the maximum environmental temperature set at 28 °C. To study the effect of multiple infections caused by different bacterial pathogens, 3 strains were chosen based on biochemical and molecular characterization, as representatives of the identified species (1N3, 2N5, and 3N1). The selected strains were inoculated in rosemary in all pairwise combinations and in combination with all 3. Multiple inoculations were performed at each inoculation point by placing 10 µL of a suspension containing 10^8^ CFU/mL of each strain. Then, all inoculated plants were kept in the glasshouse under the conditions described above. Disease severity (S) was recorded using the following disease symptom scale: 0 = no symptoms; 1 = discoloration at the inoculation point (brown or black color); 2 = browning and/or hypertrophy, presence of adventitious roots; 3 = wilted plant (**Figure 2**). S was expressed as the average of values recorded at all inoculation points for each sample. Wilting incidence (I), expressed as the percentage of wilted plants on the total inoculated plants, was also highlighted. At the end of the experiment, bacterial isolation was performed from plants inoculated with the single three strains. Purified colonies were checked for morphology and tested for HR on tobacco plants.

**Figure 3:**
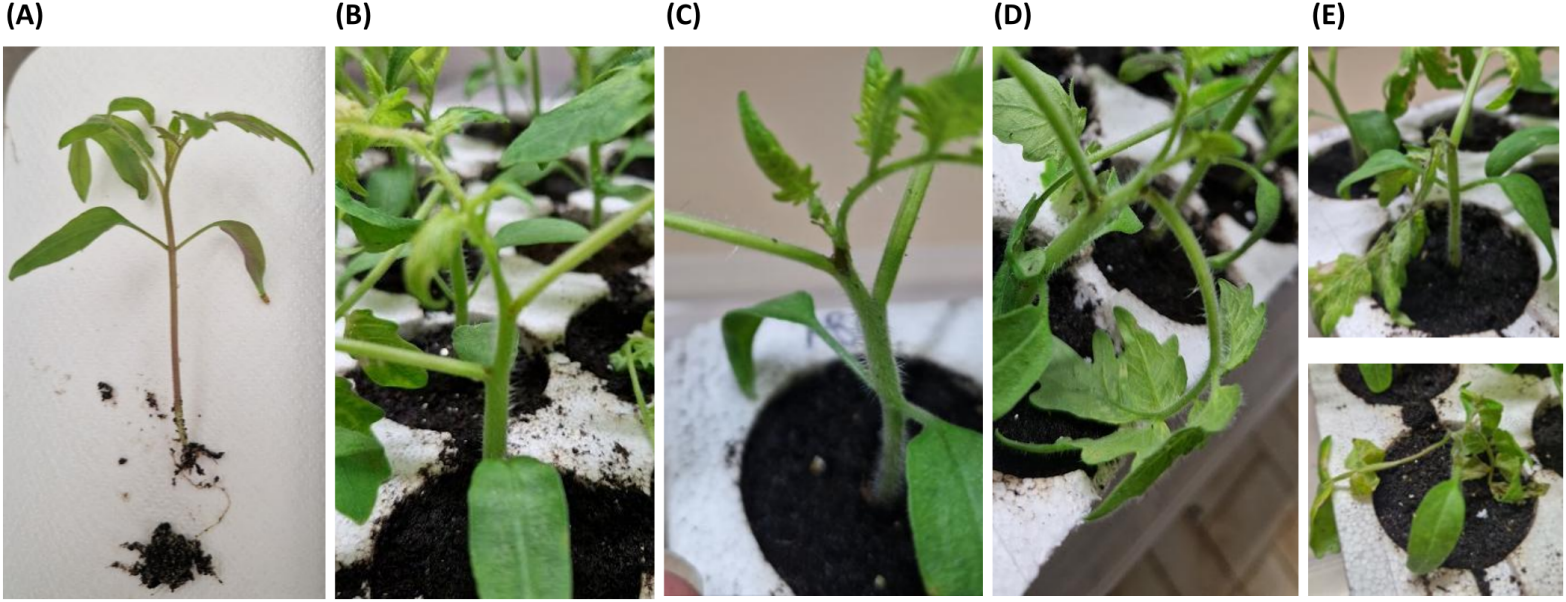
Disease scale adopted for multiple infection experiments on tomato plants. **(A)** level 0, no symptoms; **(B)** level 1, dark spot of 1 mm; **(C)** level 2, dark spot more than 1 mm; **(D)** level 3, petiole wilting; **(E)** level 4, leaf or whole plant wilting.

Multiple interactions were also studied using *Solanum lycopersicum* (variety Moneymaker) as a model host plant. To this aim, the 3 representative strains (1N3, 2N5, and 3N1) were inoculated on three-leaf stage tomato plants, at the petiole insertions of the basal and middle leaf. Each treatment was repeated on 5 plants. Single strains, all pairwise combinations and the combination with all 3 served as an inoculum: a drop of 10 µL containing a final concentration of 10^8^ CFU/mL of each bacterium was used. Moreover, in the case of pairwise combination and combination of three, the strains were inoculated in different time sequences, with an interval of 24 hours. All the combinations of 2 and 3 strains were realized by changing the order of inoculation, thus performing all the possible strain sequences; the 3 strains were also inoculated at the same time (**Supplementary Table 2**). The negative control was represented by plants inoculated with 10 µL of SDW. All inoculated plants were kept at 26 °C ± 1 and with a 12-hour photoperiod, in a climatic chamber. The disease symptoms were recorded every day for 120 hours, with the following disease scale: 0 = no symptoms; 1 = dark spot of 1 mm; 2 = dark spot more than 1 mm; 3 = petiole wilting; 4 = leaf or whole plant wilting (**Figure 3**), and the disease severity (S) was calculated as reported above.

### 2.6 Host range

Tomato (variety Moneymaker), chickpea (*Cicer arietinum*, variety Ares), pea (*Pisum sativum*, variety Lincoln), melon (*Cucumis melo*, variety Supermarket), onion (*Allium cepa*, Italian local variety Aprilatica) and lettuce (*Lactuca sativa*, variety Ninfa) were used to determine the host range of the bacterial isolates. 3 plants for each test-host were grown in a phytothrone at 26 °C for 10 days. The plant stems were punctured with a sterile needle at 3 points: above the ground, in the middle, and at the top of the plants, and then inoculated with 5 µl of each 10^8^ CFU/ml bacterial suspension at each wound. Lettuce plants were instead inoculated only above the cotyledons while onions were inoculated by spraying leaves with a bacterial suspension at a concentration of 10^8^ CFU/mL. The plants were kept in a humid chamber for 48 hours after inoculation. Negative control plants were treated with SDW. All plants were kept at 26 °C in the phytothrone at 12 hours day/night photoperiod. The symptoms on tomato, chickpea, pea, melon, and lettuce were recorded starting from the second day post-inoculation, every 2 days, for 1 week, reporting compatible and incompatible interactions on each plant. Compatible interaction was represented by superficial necrotic spot, depressed necrotic spot extending beyond the inoculation point, confluent depressed spots, and wilted plant.

### 2.7 Motility test

The swimming and swarming abilities of the strains were determined on swim and swarm agar plates, respectively, as described by Déziel et al. (2001). Plates were inoculated in the center by using a sterile toothpick. Motility was then assessed qualitatively by measuring the diameter of the turbid zone formed by the bacterial cells migrating away from the point of inoculation after 24- and 48- hours incubation at 27 °C. *Pseudomonas chlororaphis* M71 strain was used as a positive control (Raio et al., 2020).

### 2.8 Biolog assay

Biolog GenIII microplates were used for bacteria identification and to reconstruct the metabolic profiles of 3 *Pseudomonas* strains representative of each species (1N3, 2N5, and 3N1). The system evaluates the metabolic activity of each strain based on the reduction of tetrazolium violet dye in the presence of 71 different carbon sources and 23 chemical sensitivity assays. Color development in the wells was recorded by using the automated OmniLog system (Biolog) after 24 hours of incubation. Data were expressed in Biolog Units (BU) and visualized as a heatmap using the ‘*pheatmap*’ package in R (Kolde, 2019). For carbon sources, the absorbance value of the negative control well was subtracted from the absorbance values of all other wells. Wells with absorbance values below 50 BU were considered negative. For the sensitivity assays, the absorbance of the negative control was subtracted from the individual values, and each resulting value was normalized against the positive control well. A reduction of ≥70% in absorbance compared to the positive control was considered indicative of high sensitivity to the tested compound. Differences among strains were evaluated by comparing the absorbance values for each compound against the range defined by the mean ± standard deviation. Nutritional similarity was evaluated by the niche overlap index (NOI) calculated with the following formula (Wilson and Lindow, 1994)


$$NOI=\frac{NC}{N}$$

where NC is the number of carbon sources used by both bacteria, and N is the number of carbon sources used by the single bacterium.

### 2.9 Siderophore production

Quantitative analysis of siderophore production was conducted using the microplate method described by Boukelloul et al. (2024), with some modifications. For each strain, 30 µL of a bacterial suspension prepared in saline solution (OD600 = 0.1) was inoculated into 3 mL of LB broth, in triplicate. After 2 days of incubation at 28 °C under shaking, the supernatant was collected after centrifuging the cultures at 10,000 g for 10 min. A freshly prepared CAS reagent was employed for siderophore determination. The reagent was prepared by combining three different solutions, prepared separately as follows: 60.5 mg of CAS and 72.9 mg of hexadecyltrimethylammonium bromide (HDTMA) were dissolved in 50 mL and 40 mL of distilled water, respectively; a 1 mM ferric chloride (FeCl_3_‧6H_2_O) solution was prepared in 50 mL of a 10 mM HCl solution. 1 ml of the ferric chloride solution was added to 5 ml of the CAS solution and mixed slowly. A dark purple solution was obtained. The CAS-Fe solution (6 mL) was then slowly added to 4 mL of the HDTMA solution, while mixing, obtaining a dark blue color. All solutions were prepared in sterile 50 ml centrifuge tubes, using MilliQ water. 100 μL of the CAS reagent were dispensed in each well of a Corning™ Costar™ 96-Well microplate, followed by the addition of 100 µl of: i) cultures supernatants of the *Pseudomonas* strains; ii) LB broth, as a reference control; and iii) strain M71 derivative mutant M71b (Raio et al., 2020) culture supernatant, as a positive control. 8 technical replicates were prepared for each biological replicate. After incubating the supernatants in the presence of the CAS-reagent for 30 minutes, the optical density of the wells was measured at 630 nm with the Tecan Pro microplate reader. Lower absorbance values indicated greater siderophore production. The percentage siderophore units (psu) were calculated according to the following equation (Boukelloul et al., 2024):


$$psu=\frac{\left(Ar-As\right)}{Ar}\times 100$$

where “Ar” is the absorbance value of the reference control and “As” is that of the cultures’ supernatant, both mixed with the CAS reagent.

### 2.10 Mutual synergistic or inhibiting effect of bacterial strains

The production of antibacterial metabolites mediating reciprocal growth inhibition was evaluated using the cross-streaking method. For each strain (1N3, 2N5, and 3N1), a bacterial suspension of 10^7^ CFU/mL was prepared, and 50 μL were uniformly spread over one half of a TSA plate. Plates were incubated at 27 °C for 48 h to allow the production of antibacterial compounds and their diffusion through the agar medium. Subsequently, 5 μL of freshly prepared bacterial suspensions were streaked perpendicularly to the pre-grown strain (tester strain) using a sterile loop; control plates were also streaked in the absence of the tester bacterium. Plates were incubated at 27 °C for an additional 48 h. Antagonistic activity was assessed as a reduction or complete inhibition of target strain growth compared to the corresponding control plates. Another type of antagonism assay was conducted in a liquid medium. The three strains were grown on Ayers Mineral Salt Medium (NH_4_H_2_PO_4_, 1 gr/L; KCl, 0.2 gr/L; MgSO_4_‧7H_2_O, 0.2 gr/L; pH = 7.2) amended with 0,2% glucose for 48 h at 27 °C (Schaad et al., 2001). Cultures were centrifuged at 8000 rpm for 10 minutes, and the supernatants were sterilized by filtration (Millipore 0.2 µm filters). 2 mL of the supernatant from a strain culture were mixed with 2 mL of Ayers medium (Schaad et al., 2001) supplemented with 0,1% glucose and inoculated with 40 µL of a bacterial suspension (OD600 = 0.1) from one of the other two strains. These new cultures were incubated at 27 °C for 2 and 5 days under shaking. The optical density (600 nm) of all the obtained culture combinations was measured and compared with that reached by each bacterium grown in the non-inoculated medium. The experiment was performed in triplicate.

### 2.11 Biofilm production by bacterial strain combinations

The selected 1N3, 2N5, and 3N1 strains were grown overnight in NB at 28 °C. The cultures were then centrifuged at 8000 rpm for 10 minutes and diluted to an OD600 = 0.02 in NB. 150 μl of each bacterial suspension were placed in the wells of microplates and incubated at 28 °C for 24, 48, and 72 h. Bacterium combinations were performed by mixing equal volumes of the single suspensions. After incubation, suspensions were discarded, then a 0.1% crystal violet solution was added to the wells, and plates were incubated at room temperature for 20 minutes. Plates were then washed with 150 µL/well of sterile PBS to remove the excess crystal violet. Finally, 150 µL/well of 70% ethanol were added, and the absorbance at 600 nm was recorded. 4 wells were inoculated for each treatment. The experiment was repeated twice.

### 2.12 Inhibition activity of volatile organic compounds

VOCs inhibition activity was assessed for the 3 *Pseudomonas* representative strains 1N3, 2N5, and 3N1. The strains were grown on TSA plates for 36 hours at 27 °C, then a suspension at an OD_600_ = 0.1, corresponding to 10^8^ CFU/ml, was prepared for each strain, using an Easyspec spectrophotometer (Safas, Monaco, France). Each strain was tested for VOC inhibitory activity against the other 2 strains using split plates containing TSA medium. On one side, 50 μL of the OD600 = 0.1 suspension were spread; 48 hours later, on the other side, and after preparing the serial dilutions of the 2 different strains to be checked, 5 drops of 20 μL from the dilution 10^–5^ were spotted. 2 plates were prepared for each strain combination, and 2 additional plates were used for single-strain controls. Plates were sealed with 2 layers of laboratory film and incubated at 27 °C. Colonies from the drop plating were counted after 24- and -48 hours, and the VOCs inhibition activity was assessed by comparing the CFUs of treated plates with their corresponding controls. Inhibition was expressed as a reduction in the number of colonies compared to the control.

### 2.13 Statistical analyses

Statistical analyses were performed using PAST software v.5.2.1 and GraphPad Prism v.10.1. Disease severity data were analyzed using the non-parametric Kruskal–Wallis test followed by Dunn’s *post hoc* test. All other statistical analyses were performed using one-way ANOVA followed by Tukey’s HSD *post hoc* test or using the Kruskal–Wallis test followed by Dunn’s *post hoc* test when assumptions of normality and homogeneity of variances were not met. Unpaired *t*-test was applied in the VOCs data statistical analysis.

## 3 Results

### 3.1 Bacteria isolation and characterization

No fungal colonies were obtained from the roots and collar of rosemary plants on PDA medium. Bacterial colonies exhibiting different morphologies were instead constantly isolated on NGA plates. After purification, 8 isolates representative of the different colony morphologies were characterized and identified (**Supplementary Figure 2**). All strains were Gram-negative as indicated by the KOH test. Strains 1N3, 1N4, 2N5, and 2N9 were fluorescent on KB agar medium, while 1N2, 2N8, 3N1, and 3N3 were not fluorescent; additionally, the last two strains exhibited mucoid colonies with a marked yellow color. LOPAT tests revealed two distinct profiles among the eight strains: profile I included 1N2, 1N3, 1N4, 3N1, and 3N3 strains, which induced potato rot and tobacco HR. This profile corresponds to the LOPAT profile of *Pseudomonas viridiflava*. However, strain 1N3 and 1N4 were fluorescent on KB agar, as atypical *P. viridiflava* described in a previous work (Cariddi et al., 2024). Profile II included 2N5 and 2N9 strains, which were only positive for HR induction on tobacco plants (**Table 1**). 2N8 strain profile could not be assigned.

**Table 1:** Fluorescence on KB agar and LOPAT test response of the eight rosemary isolates.

Strain	Fluorescence on KB	LOPAT					
		Levan	Oxidase	Potato rot	Arginine dihydrolase	Tobacco HR	Interpretation
1N2	–	–	–	+	–	+	*P. viridiflava*
1N3	+	–	–	+	–	+	*P. viridiflava*
1N4	+	–	–	+	–	+	*P. viridiflava*
2N5	+	–	–	–	–	+	*P. syringae* pv. *savastanoi*
2N9	+	–	–	–	–	+	*P. syringae* pv. *savastanoi*
2N8	–	–	–	–	–	+/-	NLP
3N1	- (yellow colony)	–	–	+	–	+	*P. viridiflava*
3N3	- (yellow colony)	–	–	+	–	+	*P. viridiflava*

-, negative reaction; +, positive reaction; +/- weak reaction; NLP, no LOPAT profile detected.

### 3.2 Identification and phylogenetic affiliation of bacterial strains

Based on metabolic profile analysis performed using Biolog GENIII microplates, three different *Pseudomonas* species were identified. Strains 1N2, 1N3, 1N4, and 2N8 were identified as *Pseudomonas syringae* pv. *primulae*, 2N5 and 2N9 as *Pseudomonas cichorii*, while 3N1 and 3N3 as *Pseudomonas viridiflava*. Results of identification are reported in **Table 2**.

**Table 2:** Identification of the eight rosemary isolates obtained by analysis of metabolic profiles performed by Biolog GENIII microplates.

Strain	Identification	Probability	Similarity	Distance
1N2	*P. syringae* pv. *primulae*	0.971	0.620	5.230
1N3	*P. syringae* pv. *primulae*	0.646	0.646	5.096
1N4	*P. syringae* pv. *primulae*	0.774	0.511	4.908
2N5	*Pseudomonas cichorii*	0.617	0.617	5.509
2N9	*Pseudomonas cichorii*	0.617	0.617	5.509
2N8	*P. syringae* pv. *primulae*	0.674	0.674	4.719
3N1	*Pseudomonas viridiflava*	0.796	0.537	4.653
3N3	*Pseudomonas viridiflava*	0.674	0.674	4.637

Biolog results differ from MLSA identifications because *P. alliivorans* and *P. asturiensis* are not included in the Biolog database. MLSA results were considered the definitive identifications of the species.

Molecular identification based on sequencing of the 16S rDNA region assigned isolates 1N2, 1N3, and 1N4 to *P. alliivorans*, isolates 2N5 and 2N9 to *P. asturiensis*, and isolates 2N8, 3N1, and 3N3 to *P. viridiflava*, with identity values ranging from 99.66% to 100% (**Table 3**). The molecular identification based on *gyrB*, *rpoB*, and *rpoD* genes further supported this distribution into three phylogenetic groups. Specifically, isolates 1N2, 1N3, and 1N4 showed their highest similarity to *P. viridiflava* and *P. syringae* pv*. primulae* in housekeeping genes, with identity values higher than 97% only for the *rpoB* gene. Isolates 2N5 and 2N9 consistently matched *P. asturiensis* across all loci, except for the *rpoB* gene, while isolates 2N8, 3N1, and 3N3 clustered robustly with *P. viridiflava*, displaying high identity values (>97%) in all sequenced regions (**Table 3**).

**Table 3:** Molecular identification of the eight bacterial isolates based on 16S rDNA, *gyrB*, *rpoB* and *rpo*D regions sequencing.

Strain	16S rDNA (ID%)	*gyrB* (ID%)	*rpoB* (ID%)	*rpoD* (ID%)
1N2	*P. alliivorans* (99.72%)	*P. viridiflava* (93.48%)	*P.viridiflava* (97.70%)	*P. syringae* pv. *primulae* (96.68%)
1N3	*P. alliivorans* (99.74%)	*P. viridiflava* (93.37%)	*P. viridiflava* (97.55%)	*P. syringae* pv. *primulae* (96.68%)
1N4	*P. alliivorans* (99.73%)	*P. viridiflava* (93.59%)	*P. viridiflava* (98.02%)	*P. syringae* pv. *primulae* (96.63%)
2N5	*P. asturiensis* (100%)	*P. asturiensis* (95.55%)	*P. syringae* (97.06%)	*P. asturiensis* (96.31%)
2N9	*P. asturiensis* (99.72%)	*P. asturiensis* (95.42%)	*P. syringae* (97.06%)	*P. asturiensis* (96.31%)
2N8	*P. viridiflava* (100%)	*P. viridiflava* (99.51%)	*P. viridiflava* (98.99%)	*P. viridiflava* (99.85%)
3N1	*P. viridiflava* (100%)	*P. viridiflava* (99.82%)	*P. viridiflava* (99.24%)	*P. viridiflava* (97.89%)
3N3	*P. viridiflava* (99.66%)	*P. viridiflava* (99.83%)	*P. viridiflava* (99.04%)	*P. viridiflava* (97.92%)

The BLASTn tool (http://blast.ncbi.nlm.nih.gov/) was used to identify the most similar sequences in the NCBI nucleotide database, with the “sequences from type material” option enabled. In brackets, the percentage of identity (ID%).

**Figure 4:**
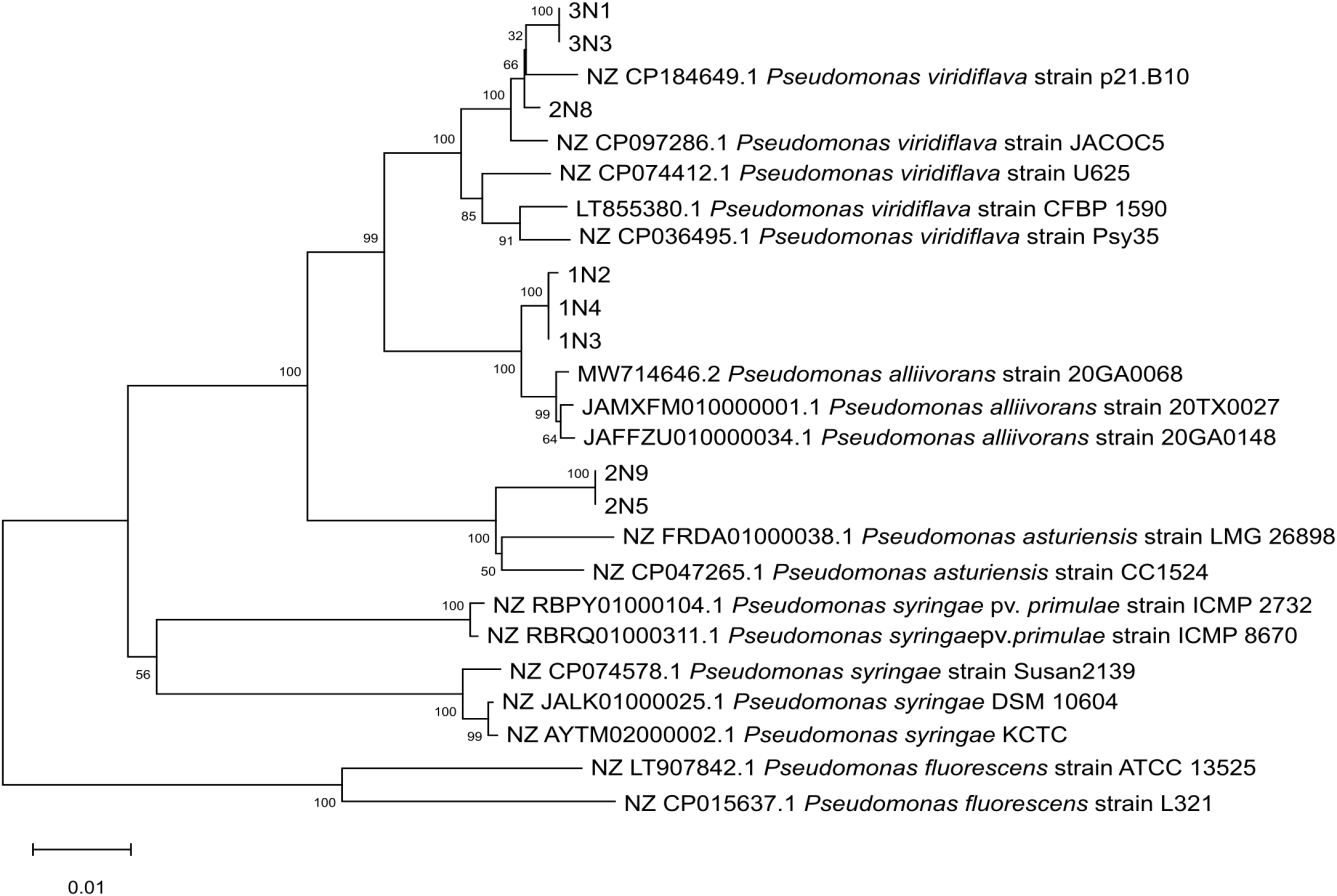
Phylogenetic tree obtained through the MLSA (2365 bp). The percentage of replicate trees in which the associated taxa clustered together in the bootstrap test (1000 replicates) is shown next to the branches. Trees are drawn to scale, with branch lengths expressed in the same units as those used to compute evolutionary distances. Distances are reported in units of base substitutions per site.

The phylogenetic analysis based on the MLSA consistently grouped the *Pseudomonas* isolates into three distinct clades. Strains 2N8, 3N1, and 3N3 (the latter two having the same concatenated sequence), grouped with *P. viridiflava* species. Isolates 1N2, 1N3, and 1N4 clustered with *P. alliivorans* species, with 1N3 and 1N4 sharing the same nucleotide sequence. This species formed a separate monophyletic clade distinct from *P. viridiflava* (its closest neighbor). Finally, 2N5 and 2N9, also sharing the same nucleotide sequence, grouped with the two *P. asturiensis* species (**Figure 4**), another member of the *P. syringae* group, with *P. viridiflava* again representing the closest relative. Phylogenetic trees obtained for individual genes are provided in **Supplementary Materials** (**Supplementary Figures 3–6**).

### 3.3 Pathogenicity test and co-infection in rosemary plants

Pathogenicity of strains 1N3, 2N5, and 3N1, chosen as representatives of *P. alliivorans*, *P. asturiensis*, and *P. viridiflava* species respectively, was assessed on healthy rosemary plants by recording the disease severity (S) and wilting incidence (I). The effect of bacterial co-infection was then evaluated by inoculating the strains in pairs and all three together. As a result, *P. alliivorans* 1N3 caused the heaviest symptoms, represented by a complete wilting in 83.3% plants; moreover, wilting was observed in plants inoculated with 1N3 + 3N1, 2N5 + 3N1, and 1N3 + 2N5 + 3N1 at a rate of I = 33.3%, 60.0%, and 20.0%, respectively. Disease severity (S) was calculated for the three strains inoculated at the different combinations. The heaviest symptom expression was observed after inoculation of 1N3 individually (S = 2.39 ± 1.01), which was significantly different from 2N5 (S = 0.61 ± 0.49) and 3N1 (S = 0.89 ± 0.46) strains. Co-inoculation of 2N5 and 1N3 strains significantly reduced the S value (S = 0.80 ± 0.75), indicating that the 2N5 strain may have partially inhibited the virulence of 1N3. All other combinations of co-inoculated strains did not induce significant changes in symptom development on rosemary plants. The S average values are reported in **Table 4**. Successful isolations of bacteria were achieved from single-strain infected plants, and the three reisolated strains showed a colony morphology comparable with the inoculated strains and all resulted HR positive in tobacco.

**Table 4:** Disease severity recorded on rosemary plants after inoculation with *Pseudomonas alliivorans* 1N3, *P. asturiensis* 2N5, and atypical *P. viridiflava* 3N1.

Strain	Disease severity (S)
1N3	2.39 ± 1.01 a
2N5	0.61 ± 0.49 d
3N1	0.89 ± 0.46 cd
1N3 + 2N5	0.80 ± 0.75 cd
1N3 + 3N1	1.59 ± 1.29 bc
2N5 + 3N1	2.07 ± 1.24 ab
1N3 + 2N5 + 3N1	1.33 ± 1.07 bcd
Negative control	0 e

Data represent the average of symptom values ± standard deviation. Disease severity was recorded according to an arbitrary disease symptom scale: 0, no symptoms; 1, discoloration at the inoculation point (brown or black color); 2, browning and hypertrophy, presence of adventitious roots; 3, plant wilting. Data were analyzed by the non-parametric Kruskal-Wallis test. Data followed by different letters were statistically different (p ≤ 0,05).

### 3.4 Pathogenicity test and co-infection in tomato plants

**Figure 5:**
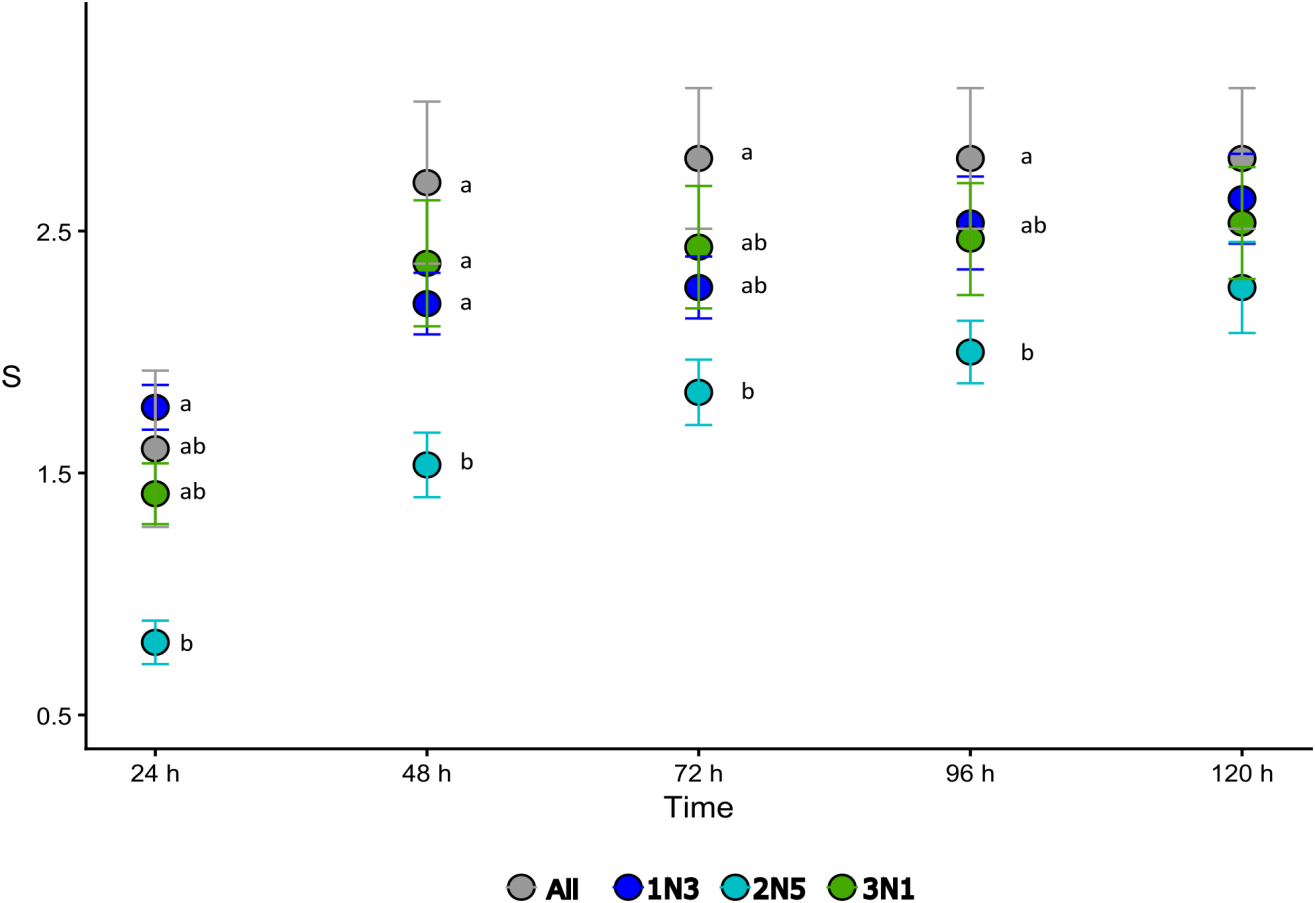
Progression of disease severity over time in plants treated with single bacterial strains and their combination (All). Disease severity (S) was assessed at each time point and is reported as mean ± standard error (SE). Statistical differences were evaluated using a Kruskal–Wallis test followed by Dunn’s *post hoc* test. Different letters indicate statistically significant differences among conditions within the same time point (p < 0.05).

The pathogenicity of the three representative strains, inoculated individually or in combinations, was assessed on healthy tomato plants as described in the section “Materials and Methods”. Among the single-strain inoculations, *P. alliivorans* 1N3 and *P. viridiflava* 3N1 produced the strongest symptoms up to 120 h. In contrast, inoculation with *P. asturiensis* 2N5 resulted in a delayed symptom development, with S values significantly lower than those observed for both 1N3 and 3N1 during the first 48 h after the initial inoculation. When all three strains were inoculated simultaneously (All), the resulting S index exceeded that of single-strain inoculations; however, it was not significantly different from 1N3 or 3N1 alone, while remaining significantly higher than 2N5 up to 96 hours after inoculation (**Figure 5**).

**Figure 6:**
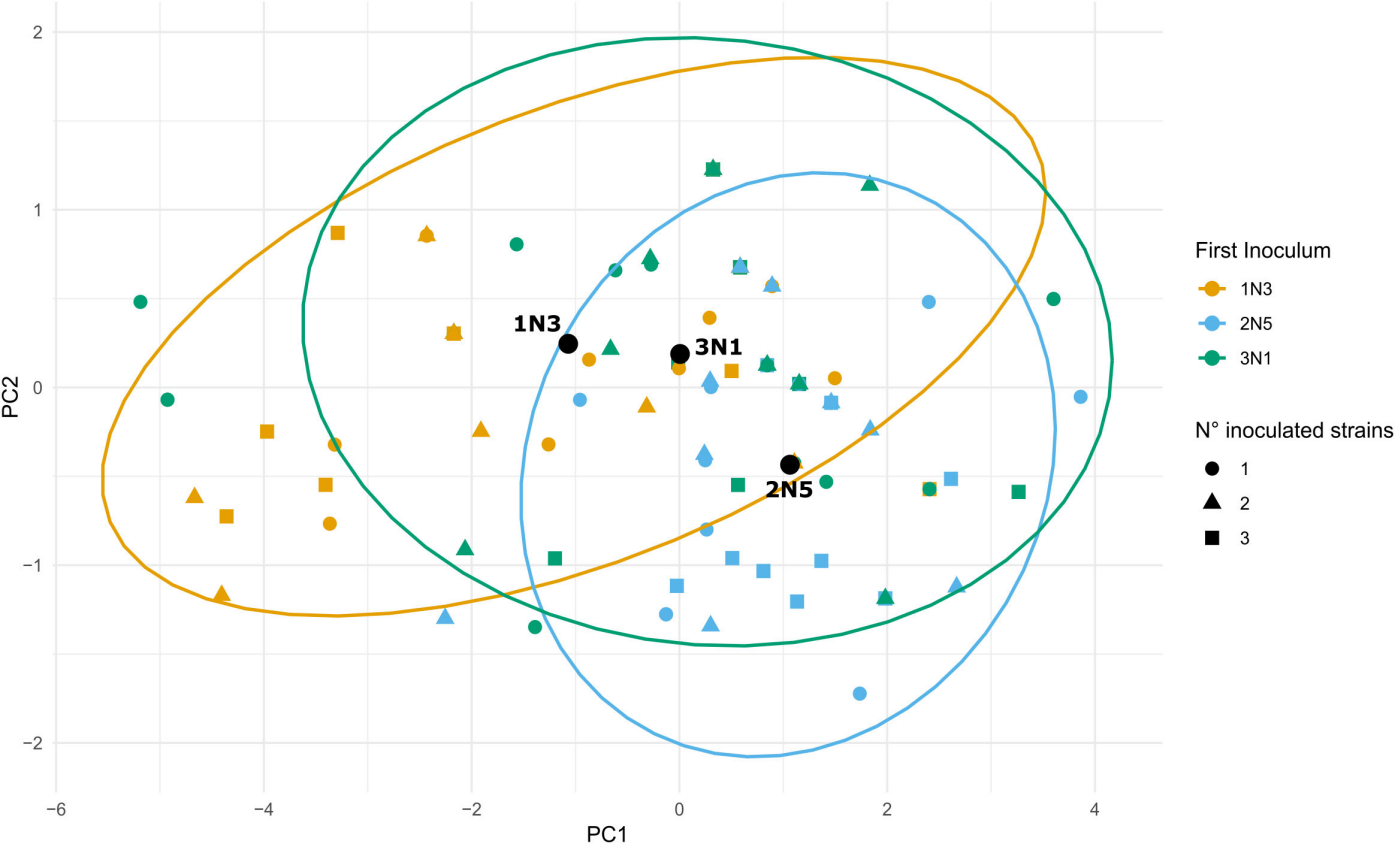
Principal component analysis (PCA) of disease severity dynamics across inoculation treatments. The PCA was performed using S measurements collected at 24, 48, 72, 96, and 120 hours post-inoculation. Each point represents an individual plant, colored by the first inoculated strain and shaped according to the total number of strains applied. Ellipses depict 95% confidence regions for groups defined by the first inoculum, with group centroids shown as filled black points. The PCA was constructed in R using the *ggplot2* package.

Concerning sequential inoculations, disease severity was strongly affected by the inoculation order, with combinations in which 1N3 was applied first being the most virulent, followed by 3N1-first and 2N5-first combinations. High competition for infection site occupation or induction of systemic resistance in the host can be considered as possible mechanisms implemented by 1N3 strain. The principal component analysis (**Figure 6**) based on scaled S values across all time points confirmed that treatment separation was driven by the first inoculated strain (PERMANOVA *p* = 0.001; MANOVA *p* = 1.805 × 10^-7^), with 1N3-first and 2N5-first groups separating along PC1 and PC2, and 3N1-first group occupying an intermediate position.

In general, disease severity increased over time in plants inoculated with single strains, whereas it reached a more stable level in plants treated with strain combinations (**Supplementary Table 3**; raw data is provided in the **Data Sheet 1** of **Supplementary Materials**). Symptoms caused by the three strains inoculated simultaneously (All) or at 24-hour intervals appeared similar when 1N3 was inoculated first at all time points. Indeed, the 1N3 + 3N1, 1N3 + 3N1 + 2N5, and 1N3 + 2N5 + 3N1 combinations consistently exhibited the highest S values. All the other three-strain combinations’ S values (3N1 + 2N5 + 1N3, 3N1 + 1N3 + 2N5, 2N5 + 3N1 + 1N3, and 2N5 + 1N3 + 3N1) were lower. In general, strain 2N5 reduced the aggressiveness of the disease of 1N3 and 3N1 strains alone, suggesting a putative antagonistic effect. However, the S of 2N5 + 1N3 and 2N5 + 1N3 + 3N1-treated plants, in which 1N3 was inoculated as the second strain, increased steadily from 96 hours onward; in particular, at 120 hours, 2N5 + 1N3 was significantly higher than 2N5 + 3N1 + 1N3, which exhibited the lowest S value. Other statistically significant differences are reported in **Supplementary Figure 7**, where all single-strain treatments and strain combinations are shown together for each time point, allowing direct comparison of symptom progression among treatments at late stages of infection.

### 3.5 Host range

Results of pathogenicity tests on tomato, pea, chickpea, melon, lettuce, and onion revealed polyphagous behavior of the strains isolated from rosemary plants with a different level of severity on species, ranging from the appearance of necrotic spots at the inoculation point to deepened damage in the plant tissues until the complete collapse of the stem with consequent plant wilting. Strains 1N3 and 3N1 were able to induce symptoms on the six tested plant species, while 2N5 did not infect onion plants.

### 3.6 Siderophore production and motility tests

**Figure 7:**
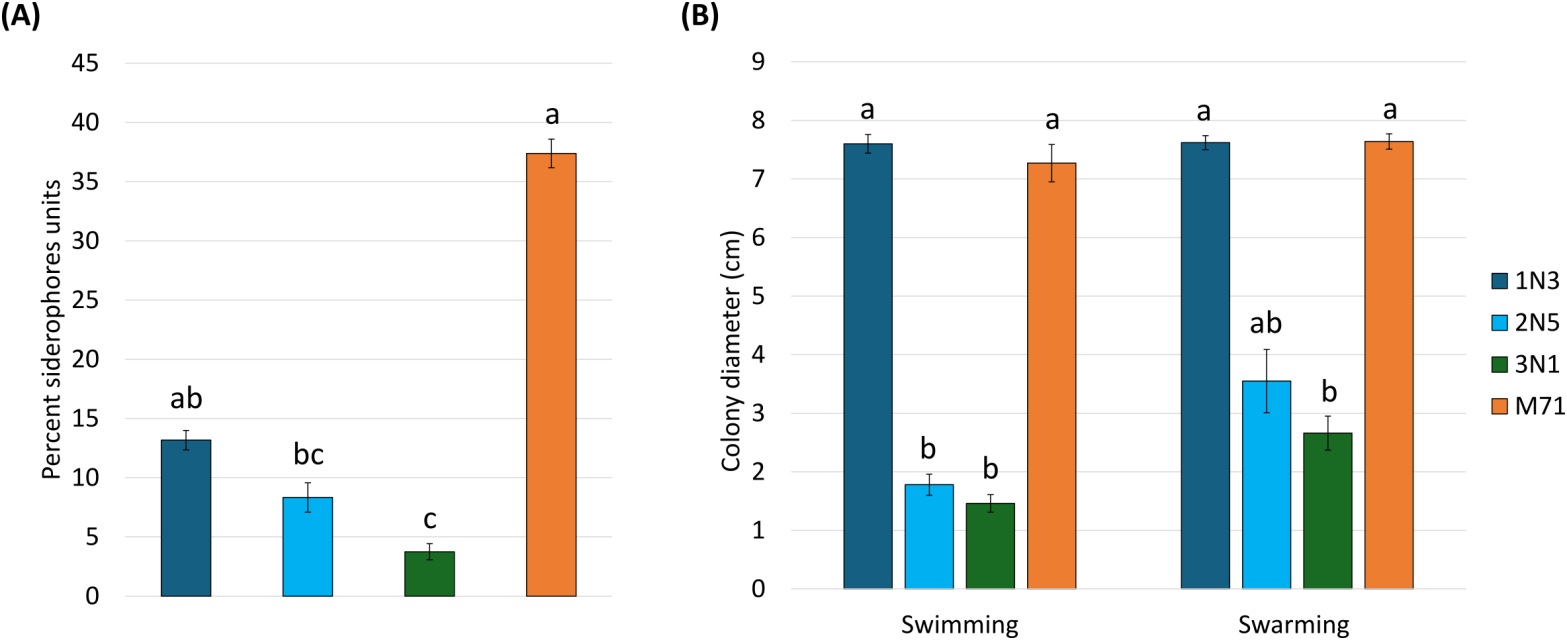
**(A)** Siderophore production expressed in percent siderophore units and **(B)** swimming and swarming abilities expressed as mean values of colony diameter (in cm). Error bars represent standard error with different letters indicating statistically significant differences (*p* < 0.05) based on one-way ANOVA followed by Tukey’s HSD *post hoc* test (or Kruskal–Wallis with Dunn’s *post hoc* test if normality and homogeneity of variances assumptions were not met).

The three *Pseudomonas* species showed different abilities to synthesize siderophores. In fact, strain 1N3 exhibited the highest siderophore production (13.17 ± 0.82 psu), followed by strain 2N5 (8.33 ± 1.24 psu) and 3N1 (3.75 ± 0.60 psu) (**Figure 7A**). Concerning bacterial motility, only strain 1N3 showed marked swimming and swarming abilities, comparable to the positive control. The other two strains (2N5 and 3N1) had reduced motility. Values of colony diameter were statistically similar for these two strains and significantly different from the M71 and 1N3 strains. Results recorded after 48 h of incubation are reported in **Figure 7B**.

### 3.7 Biofilm production by the different bacterial strain combinations

**Figure 8:**
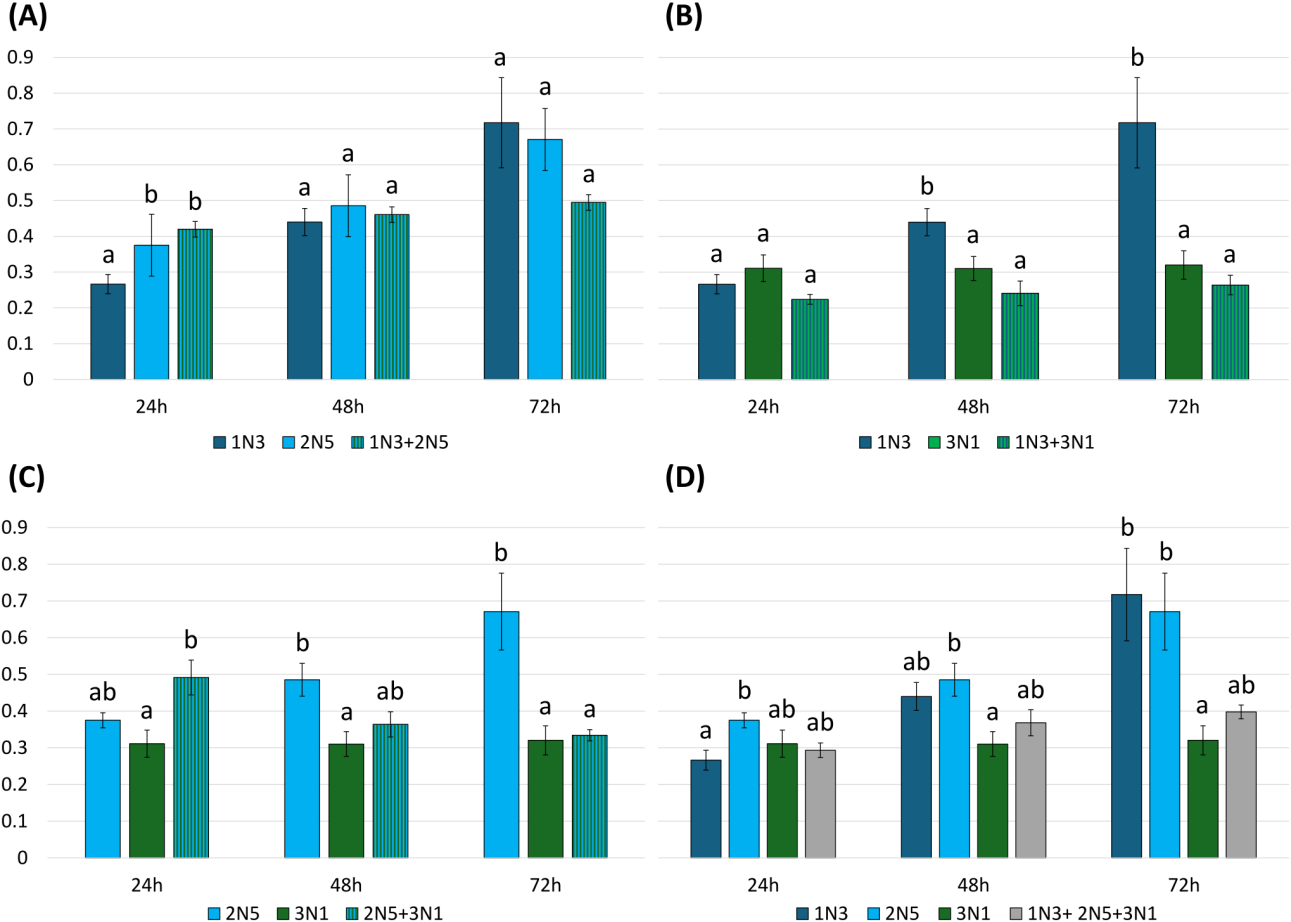
Effect of co-cultivation of *P. alliivorans* (1N3), *P. asturiensis* (2N5) and *P. viridiflava* (3N1) on biofilm production. **(A)** 1N3 + 3N1; **(B)** 1N3 + 2N5; **(C)** 3N1 + 2N5; **(D)** 1N3 + 2N5 + 3N1. Mean absorbance values at 600 nm measured after 24, 48, and 72 h of incubation are reported. Error bars represent the standard error. Statistical differences at each time point were assessed using ANOVA followed by Tukey’s *post-hoc* test, or by Kruskal–Wallis followed by Dunn’s *post-hoc* test when assumptions of normality and homogeneity of variances were not met (*p* < 0.05).

The combined cultivation of 1N3, 2N5, and 3N1 *Pseudomonas* strains had a general negative impact on biofilm production. In fact, a mutual inhibitory effect was observed for different pairwise combinations, while no synergistic interactions were recorded. Biofilm production by strains 1N3 and 2N5 remained unchanged when the two strains were co-cultivated (**Figure 8A**), whereas biofilm formation by strain 1N3 was significantly reduced in the presence of strain 3N1, starting from 48 hours of incubation (**Figure 8B**). Similarly, biofilm production by strain 2N5 was significantly lower when co-cultured with strain 3N1 after 72 hours (**Figure 8C**). No significant differences were detected when comparing the biofilm production of every single strain to that observed for the three-strain co-culture (**Figure 8D**).

### 3.8 Phenotype microarray analysis

**Figure 9:**
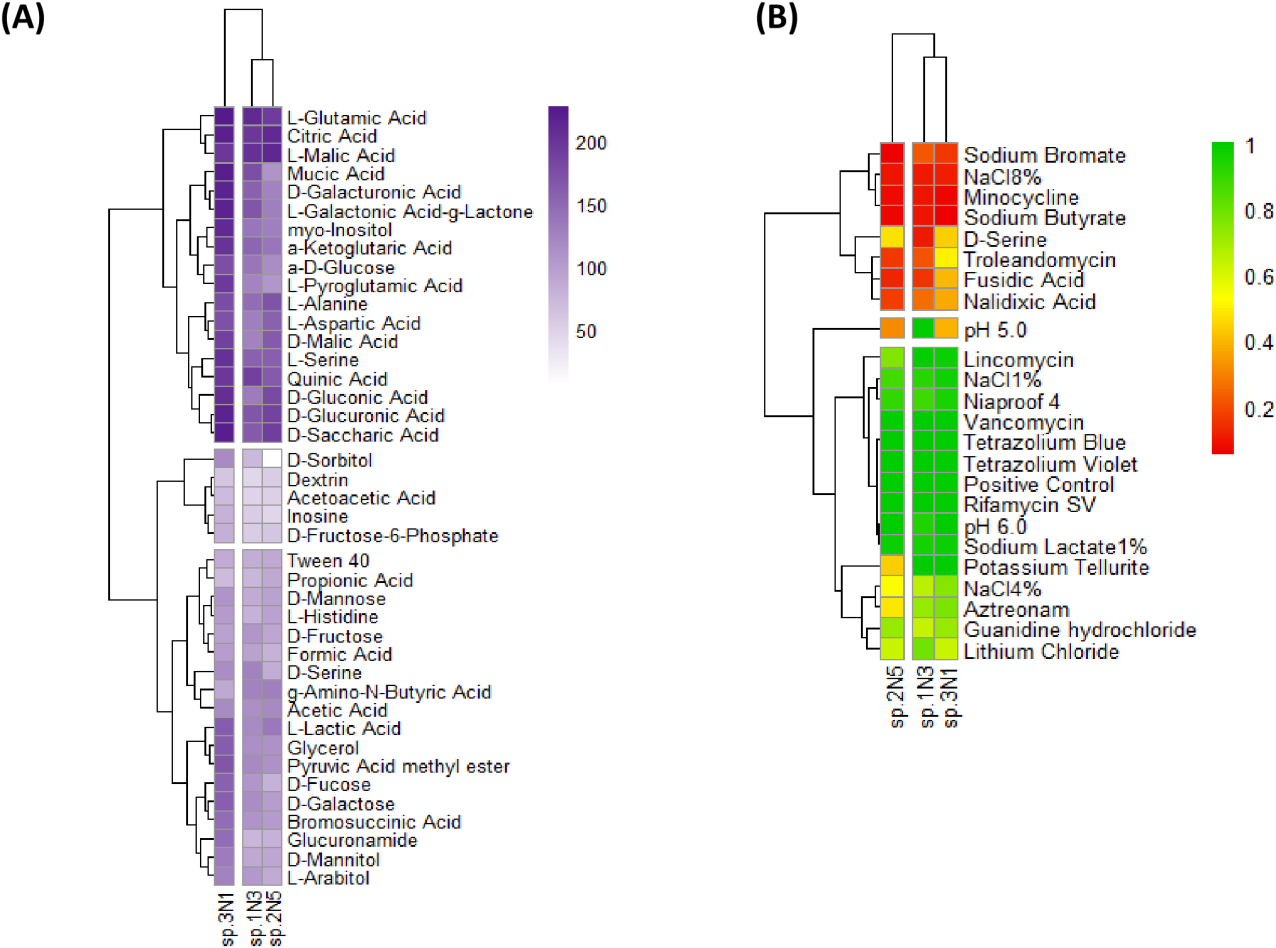
Heatmaps showing **(A)** the utilization of carbon sources and **(B)** the results of chemical sensitivity tests for the three *Pseudomonas* species. Metabolic activity on carbon sources is expressed in Biolog units (BU), with higher absorbance values indicating greater metabolic activity. Sensitivity test results are normalized on a scale from 1 (green, growth comparable to the positive control) to 0 (red, no growth in the presence of the tested compound).

The results obtained through Phenotype Microarray are reported in **Figure 9**. Concerning carbon sources (**Figure 9A**), compounds with absorbance values below 50 BU for all three strains were excluded from the analysis (**Supplementary Text 1**). Clear differences emerged among the three metabolic profiles. Strain 3N1 exhibited the highest metabolic versatility, being able to utilize a wide array of sugars (e.g., α-D-glucose, D-mannose, D-galactose), alcohols (e.g., sorbitol, mannitol), and carboxylic acids (e.g., L-lactic acid, α-ketoglutaric acid, pyruvic acid methyl ester). Strain 1N3 showed a particularly high activity toward D-fructose, but reduced metabolism of L-alanine, D-gluconic acid, and D-malic acid, compared to the other two strains. In contrast, strain 2N5 preferentially metabolized L-malic acid and propionic acid, while displaying lower activity for D-serine, mucic acid, and quinic acid, compared to the other metabolic profiles. Hierarchical clustering based on carbon source utilization revealed that strains 1N3 and 2N5 grouped together, while 3N1 formed a separate cluster. NOI index revealed a high level of nutritional compatibility (≥0.90) for all the tested two-by-two combinations, as reported in **Supplementary Table 4**.

Conversely, sensitivity tests (**Figure 9B**) highlighted a more similar behavior for strains 1N3 and 3N1, when compared to 2N5. All three strains were highly sensitive to NaCl 8%, nalidixic acid, minocycline, sodium butyrate, and sodium bromate, while strain 3N1 was less inhibited by fusidic acid, troleandomycin, and nalidixic acid compared to the other two strains. Moreover, strain 2N5 displayed a reduced growth in the presence of potassium tellurite, NaCl 4%, and aztreonam, while strain 1N3 was the only one able to grow at pH=5 and was the most inhibited by D-serine.

### 3.9 Mutual synergistic or inhibiting effect of bacterial strains

**Figure 10:**
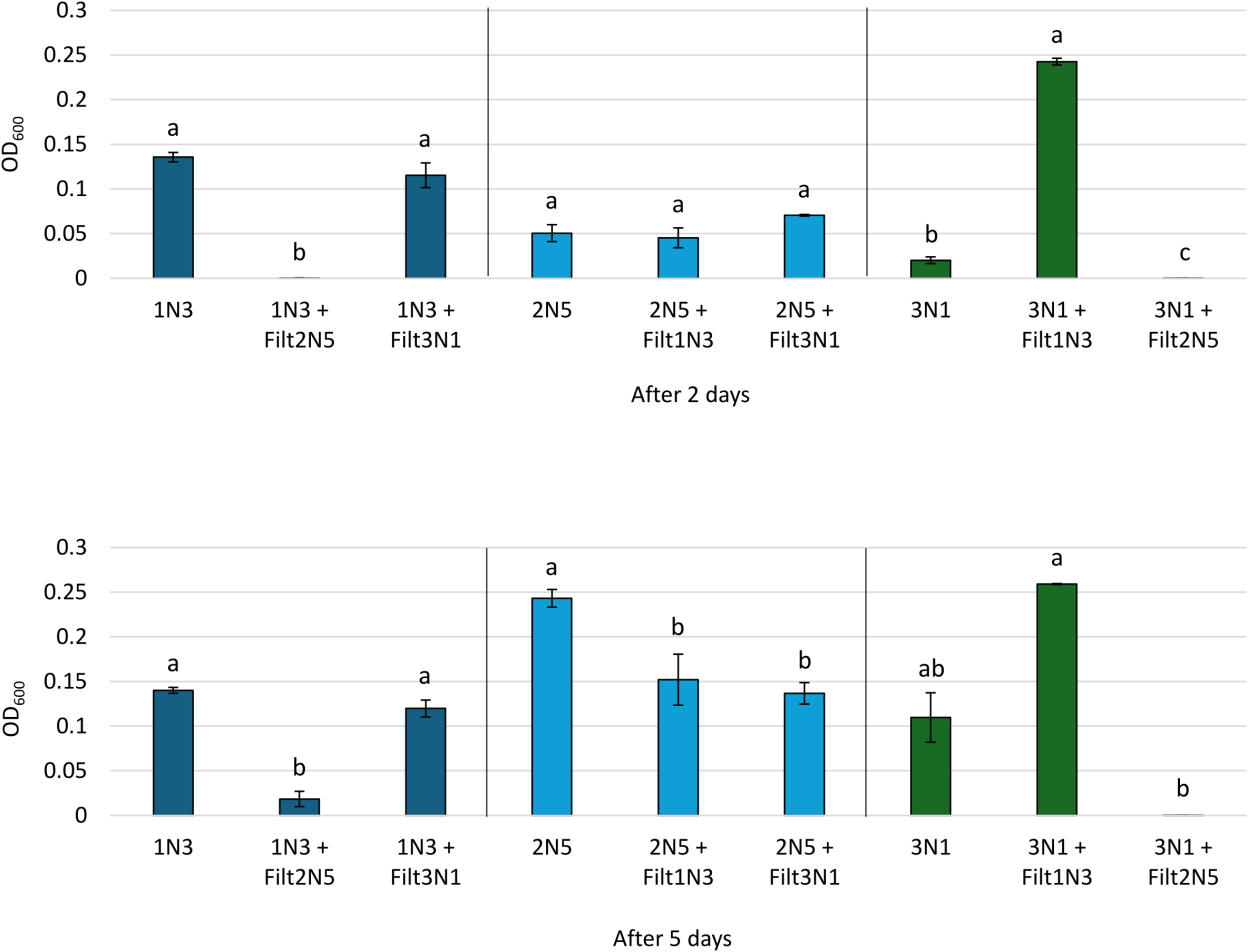
Bacterial growth of the *Pseudomonas* species expressed as OD_600_ in the absence or in the presence of the culture filtrates (Filt) obtained from the other bacteria. Error bars represent standard errors, with different letters indicating statistically significant differences (p < 0.05) based on one-way ANOVA followed by Tukey’s HSD *post hoc* test (or Kruskal–Wallis with Dunn’s *post hoc* test if normality and homogeneity of variances assumptions were not met).

Cross-streaking results, performed on the nutrient-rich growth medium TSA, revealed the absence of any bacterial growth inhibition (data not shown). Therefore, we also performed an experiment aimed at verifying the possible mutual synergistic or inhibitory effects among the three *Pseudomonas* species in a minimal liquid medium, which is known to induce secondary metabolism. The culture supernatants exhibited a more pronounced effect on the bacteria’s growth after 5 days of incubation (**Figure 10**). In particular, the culture filtrate of strain 1N3 had an inhibiting effect on strain 2N5, while promoting the growth of strain 3N1; the spent medium obtained from strain 2N5 significantly inhibited the growth of the other two bacteria; finally, strain 3N1 culture supernatant did not affect the growth of strain 1N3 but inhibited the growth of strain 2N5 after 5 days. The discrepancy between the two tests may be related to the different growth media used. To summarize, strain 2N5 emerged as the most competitive strain, effectively inhibiting the growth of both other bacteria; moreover, it was able to grow in the presence of the spent media obtained from the other two strains, although to a lesser extent. Strains 1N3 and 3N1 appeared to be compatible, as neither culture supernatant inhibited the growth of the opposite strain; in fact, the spent medium obtained from strain 1N3 had a positive effect on the growth of strain 3N1, both after two and five days.

### 3.10 Inhibition activity of VOCs

**Figure 11:**
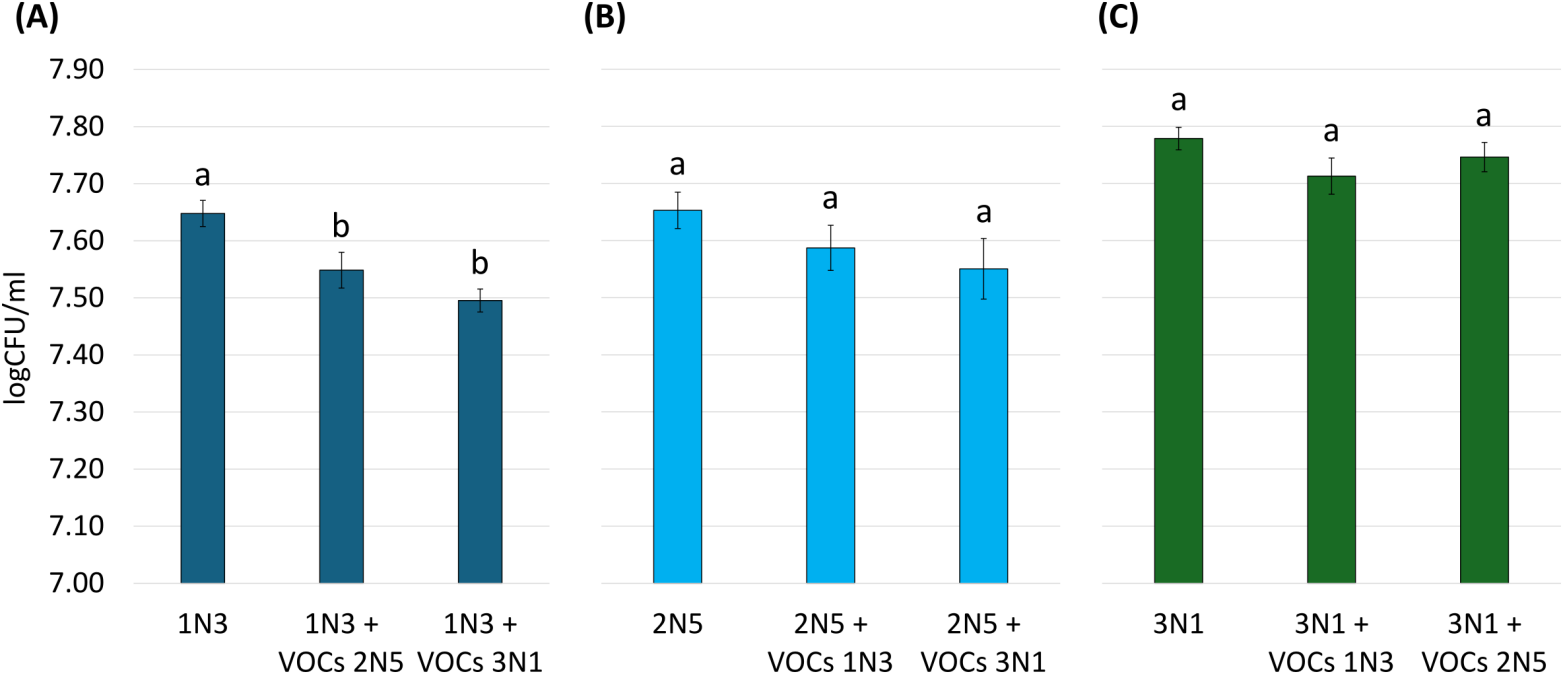
VOCs inhibitory activity. **(A)** CFU/mL of 1N3 in the absence and in the presence of VOCs from 2N5 and 3N1. **(B)** CFU/mL of 2N5 in the absence and in the presence of VOCs from 1N3 and 3N1. **(C)** CFU/mL of 3N1 in the absence and in the presence of VOCs from 2N5 and 1N3. Statistically significant differences at p < 0.05 (treatment *vs* control) were determined by unpaired *t*-test.

VOCs emitted by 2N5 and 3N1 pseudomonads had a significant inhibiting activity toward the 1N3 strain. The growth of 2N5 and 3N1 strains was instead not affected by the VOCs emitted by 1N3 and 3N1 and by 1N3 and 2N5 respectively (**Figure 11**).

## 4 Discussion

In the past decades, the role of the single pathogen as responsible for disease induction has been dominant in plant pathology. However, the development of new molecular tools for disease diagnosis has allowed us to demonstrate the coexistence of multiple pathogens in a single infection site, whose interaction may be synergistic (even determining symptom worsening) or antagonistic (mutual inhibiting activity) (Abdullah et al., 2017). Multi-infections caused by fungi, bacteria, and viruses are common in nature and may alter intra-host dynamics, as demonstrated in several cases (Dutt et al., 2022). Disease symptoms due to multiple infections have been described in different host plants, and recent investigations have evidenced that disease induction and evolution are often due to the action of different pathogenic species or strains (Bellah et al., 2023; Tollenaere et al., 2016). Plant diseases where more than one pathogen is involved in the infection process are commonly termed “complex” since diagnosis and subsequent control are more complicated than those induced by one (Lamichhane and Venturi, 2015).

In this work, we report a new multi-agent disease observed in wilted rosemary plants cultivated in an open field located in Southern Italy, showing heavy wilting and little knots on the branches. Pathogenic bacteria with different colony morphologies were isolated from diseased plants. A preliminary characterization evidenced that rosemary plant tissues hosted different bacterial species ascribable to the *Pseudomonas* genus. LOPAT tests, which are used to characterize phytopathogenic pseudomonads, evidenced that five strains (1N2, 1N3, 1N4, 3N1, and 3N3) were HR positive on tobacco and able to induce potato rot. This LOPAT profile is assimilable to *P. viridiflava*. A second group of strains (2N5 and 2N9) was instead only positive for HR and, according to the LOPAT scheme, 2N5 and 2N9 could be assimilated to *P. savastanoi*, while 2N8 did not match any LOPAT profile. The identification of two out of the five *P. viridiflava* strains (3N1 and 3N3) was confirmed by the BIOLOG test, while the remaining three strains were instead identified as *P. syringae* pv. *primulae*. Strains only positive at the HR test were identified as *P. cichorii*. The two strains identified as *P. viridiflava* at LOPAT tests and Biolog assimilation profiles showed an atypical morphology since colonies were mucoid, not fluorescent, and creamy yellow. Atypical *P. viridiflava* strains showing these characteristics have been previously isolated in Southern Italy, and were able to induce different symptoms on safflower, curly type lettuce, sugarloaf, and Catalogna chicory (Cariddi et al., 2024). Atypical *P. viridiflava* have also been previously isolated from bean and several weed species in Spain (González et al., 2003; Fernández-Sanz et al., 2022). In this case, bean plants were also infected by *P. syringae* pv. *phaseolicola*, whose high virulence could have displaced the less aggressive *P. viridiflava*, detected at a lower frequency (Fernández-Sanz et al., 2022). On the other hand, the relatively frequent detection of *P. viridiflava* from several weed species suggests that these spontaneous plants may be an important reservoir and source of inoculum for other crops (Fernández-Sanz et al., 2022). *P. viridiflava* is a polyphagous species, able to infect a wide range of hosts, inducing various symptoms on the aerial part of the plants, such as leaf spot, leaf blight, blossom blight, stem rot, pit necrosis, and bacterial canker (Lipps and Samac, 2022). Phylogenetic analyses clustered *P. viridiflava* within the PSC; however, it is considered an outsider due to differences in core genome, virulence genes, low average nucleotide identity, and some phenotypic features compared to other PSC members (Lipps and Samac, 2022).

*Pseudomonas* strains isolated from rosemary plants were also identified by MLSA of 16S rDNA, *gyrB*, *rpoB*, and *rpoD* regions sequencing. Based on this analysis, the rosemary strains were separated into three groups: the two atypical *P. viridiflava* and 2N8 strains clustered with *P. viridiflava* species, 2N5 and 2N9 clustered with *P. asturiensis* reference strains, while 1N2, 1N3, and 1N4 strains were grouped with *P. alliivorans*. Both *P. alliivorans* and *P. asturiensis* belong to the PSC and are closely related to *P. viridiflava*. So far, these two species have been reported only in Georgia (USA) (Zhao et al., 2022) and in Spain (González et al., 2012, 2013), respectively. *P. alliivorans* was isolated from dark-brown spots present on leaves of onion plants, while *P. asturiensis* was isolated for the first time from reddish spots on soybean and found as an epiphyte on two weed species. Since *P. alliivorans* and *P. asturiensis* species are not included in the Biolog database, rosemary strains were identified as the closest species present in the database. As *P. alliivorans* and *P. asturiensis* were never detected before, this work represents the first report of these two phytopathogenic *Pseudomonas* species in Italy.

Concerning phytopathogenic behaviors, the three strains chosen as representative of each identified species (1N3, 2N5, and 3N1) were pathogenic to rosemary plants with different degrees of disease severity. Wilting was reproduced by *P. alliivorans* 1N3, with the highest S value, while the lowest value was recorded for *P. asturiensis* 2N5. Hypertrophies were observed sporadically after inoculation with 3N1 and 2N5, but the low frequency recorded did not allow to unquestionably attribute this symptom to the tested strains. Because of the severe conditions set in artificial inoculations, several plants (mainly those inoculated with 1N3 strain) died after three months and then the pathogenicity test was considered ended. It cannot be excluded that hypertrophies could have developed in a later stage of the plants, or that those observed in the cultivation could be a non-specific response of the plant to stress factors. Indeed, knots, observed by Ashrafi et al. (2010), were found in rosemary plants affected by *Phytophthora citrophthora*, *Fusarium oxysporum,* and *Rhizoctonia solani*; however, the authors did not report knot development among the symptoms induced by pathogen inoculations. Moreover, co-inoculation of 1N3 and 2N5 resulted in a decrease of S compared to 1N3 alone, evidencing a putative antagonistic interaction of 2N5 toward it. No synergistic interactions were observed in the development of the disease in rosemary plants in all combinations of two or three strains studied. The three selected strains showed a wide host range, being able to infect at least five out of the six plant species tested.

Several examples of mixed infections by bacterial species have been reported, especially those where the synergistic interactions among the different pathogens led to symptom worsening (Canaday et al., 1991; Aiello et al., 2017; Sadhukhan et al., 2024). The most investigated and worldwide spread complex bacterial disease is the tomato pith necrosis, induced by different genera of bacteria, including *Pseudomonas* (*P. corrugata*, *P. cichorii*, *P. fluorescens*, *P. marginalis*, *P. mediterranea*, *P. putida*, *P. viridiflava*), *Pectobacter* (*P. atrosepticum*, *P. carotovorum*) (Lamichhane and Venturi, 2015; Aiello et al., 2017), and *Xanthomonas* (*X. perforans*) (Aiello et al., 2013, 2017). All these bacteria can cause pith necrosis on tomato plants alone or in association with one or more species, and the association is often related to enhanced severity of disease symptoms, as in the case of *P. fluorescens* and *P. corrugata* (Molan and Ibrahim, 2007), *P. marginalis* and *P. corrugata* (Kůdela et al., 2010), and *X. perforans* associated with different *Pseudomonas* species (Aiello et al., 2017). In this latter case, it was experimentally demonstrated that some *Pseudomonas* species increased the population density of the co-inoculated *X. perforans* and enhanced symptom severity on tomato plants (Aiello et al., 2017).

Antagonistic interactions can also take place in complex plant diseases. It was observed that bacterial growth in a mixed infection does not reflect single-strain growth, because of growth interference among the co-inoculated strains (Macho et al., 2007). Indeed, it was demonstrated that the degree of growth interference in mixed infection with different *P. syringae* strains was dependent on the dose of the inoculum, which in turn was related to the aggressiveness of the pathogen and the production of virulence factors by the co-inoculated strains (Macho et al., 2007). In the present work, tomato plants were used to study the *in vivo* interactions among the three strains using two and three species co-inoculation, compared to single inoculations. Results evidenced that *P. alliivorans* 1N3 alone and in all combinations where it was inoculated first, induced the heaviest symptoms. This observation suggests that 1N3 is the most virulent among the three species. On the other hand, *P. asturiensis* 2N5 was less virulent. Moreover, when 2N5 was inoculated first, it was able to reduce symptom severity induced by 1N3 during the first 96 h after the first inoculum. Symptom severity induced by *P. viridiflava* 3N1 was intermediate among the three strains. In the work of Sadhukhan et al. (2024), co-infection of *X. perforans*, *P. capsici*, and *X. arboricola* on tomato plants determined an increase in disease severity compared to *X. perforans* alone. However, the co-infection of two species determined a reduction of disease severity (Sadhukhan et al., 2024). These results suggest that co-infection of multiple species affects disease severity and that the outcomes depend on the interacting species (Sadhukhan et al., 2024).

Concerning the *in vivo* interaction among the three rosemary *Pseudomonas* species, competition seemed to be prevalent, while synergism was never observed on both rosemary and tomato plants. To support the hypothesis that competitive interactions exist among the three species, several biochemical and physiological traits were evaluated through *in vitro* experiments. Biolog phenotype microarray analysis revealed that the three selected strains showed different metabolic and sensitivity profiles. In particular, *P. viridiflava* 3N1 showed the highest metabolic versatility for carbon source assimilation, being able to utilize a wide array of sugars, alcohols, and carboxylic acids, forming a separate cluster according to the hierarchical analysis. Nevertheless, a high level of nutritional compatibility was observed among the three species. Strains with a high overlap between their metabolic routes are more likely to antagonize each other (Russel et al., 2017). Therefore, it is reasonable to hypothesize that competitive, rather than synergistic, interactions may have occurred among the three *Pseudomonas* species. Motility, biofilm, and siderophore production are also related to the ecological and antagonistic performances of bacterial strains (Josenhans and Suerbaum, 2001; Harrison and Buckling, 2009). Results obtained from the strain characterization for such traits confirmed in part these observations since strain *P. asturiensis* 2N5 and *P. alliivorans* 1N3 were the most active siderophore and biofilm producers; in addition, 1N3 showed marked swimming and swarming ability. Non-synergistic interaction between the strains was also demonstrated by combined biofilm test results. Indeed, biofilm experiments revealed a predominantly competitive interaction, particularly when *P. viridiflava* 3N1 was present. It is extremely complicated to explain the mechanism beyond bacterial interaction in biofilm formation: indeed, they are characterized by a very complex ecology in which physiochemical and biological parameters need to be considered (Simões et al., 2007). In the biofilm matrix, several interactions among the bacterial species are established with very different outcomes on the structure, composition, and function (Foster and Bell, 2012; Sadiq et al., 2023). Competitive effects of multiple bacterial species or strains in biofilm formation were already reported in previous studies, which revealed that when bacteria predominantly engage in competitive interactions within a biofilm matrix, antagonistic effects can be observed (Govaert et al., 2019). Moreover, Rao et al. (2005) showed that *Pseudoalteromonas truncata* produces microbial compounds capable of inhibiting biofilm formation by coexisting species. Similarly, *P. viridiflava* produces ecomycins; although they have been primarily described as antifungal compounds, these molecules, along with other lipopeptides, could also play a role in modulating biofilm formation by exogenous species (Miller et al., 1998; Chauhan et al., 2022).

Concerning *in vitro* antagonism tests, the cross-streaking method did not evidence the production of any antibacterial molecules able to diffuse in the agar medium and inhibit reciprocal growth. On the other hand, the test performed by growing each strain in the presence of the cultural filtrates obtained from the other strains generally evidenced mutual inhibition among them. This discrepancy might reflect differences in bacterial physiology and metabolite regulation under the two experimental conditions. In nutrient-rich solid media, bacteria are provided both physical and chemical protection, and global regulatory systems often repress the production of energetically costly metabolites, such as antibiotics, to maximize primary growth efficiency (Ruiz-Villafan et al., 2022; Molina-Santiago et al., 2019). Strain 2N5 was the most competitive among the three species, since it strongly inhibited the other two strains’ growth. This inhibition could be ascribed to the release of soluble metabolites in the surrounding environment. Indeed, in minimal media, the absence of organic nutrients imposes substantial stress, which is a well-known trigger for the activation of secondary metabolism (Yoon and Nodwell, 2014). Moreover, these results confirm the outcomes of pathogenicity tests on rosemary and tomato plants, where 2N5, co-inoculated with 1N3, significantly reduced the symptom severity induced by this latter strain. On the contrary, the culture filtrate obtained from strain 1N3 enhanced the growth of 3N1, whose spent media did not inhibit 1N3, suggesting the compatibility between the two strains. Under these limiting conditions, it may be possible that metabolic intermediates released by 1N3 could alleviate biosynthetic bottlenecks in 3N1, enabling faster growth and higher biomass accumulation (McKinlay, 2023). Finally, regarding the production of VOCs, 2N5 and 3N1 partially inhibited the growth of *P. alliivorans* 1N3. No other interaction due to VOCs was detected among the three strains.

In conclusion, *P. alliivorans*, *P. asturiensis*, and *P. viridiflava* strains isolated from rosemary plants were all pathogenic to this host, causing stem wilting. The retrieval of three pathogenic species evidences a case of an unreported multi-infection disease. These are complex diseases since the difficulty of evaluating the specific role of each species in pathogenesis, symptom development, and plant response to infection. Under multi-infections, pathogens may interact either directly (mechanical or chemical interactions) or indirectly through host resources or defense, and their interactions may be synergistic or antagonistic (Dutt et al., 2022). In this study, the three bacterial species were characterized for their pathogenic behavior, antagonistic properties, and mutual interactions when inoculated on rosemary and tomato plants. It was found that the three bacteria established only competitive interactions *in vivo*. The high nutritional competition, as well as the ability to produce and release soluble metabolites and VOCs, could in part explain the co-inoculation outcomes observed in rosemary and tomato plants. However, it cannot be excluded that *P. asturiensis* 2N5, inoculated first, was able to prime the host response toward *P. alliivorans* 1N3, which was the most aggressive strain when inoculated alone. This work has mainly focused on the bacteria characterization and the study of the relationships among them. However, the results obtained by the *in vivo* tests strongly suggest that investigating the plant response to each of the three *Pseudomonas* species could provide new information regarding their respective role in the onset of rosemary disease.

## Data Availability

The datasets presented in this study can be found in online repositories. The names of the repository/repositories and accession number(s) can be found below: https://www.ncbi.nlm.nih.gov/genbank/, from PX857324 to PX857331; from PX896467 to PX896490.
