# Supplementary material for: Pseudomonas alliivorans, Pseudomonas asturiensis, and Pseudomonas viridiflava multi-infections and competitive interactions in wilted plants of Rosmarinus officinalis L
Source: Front Plant Sci. 2026 Apr 15;17:1799303. doi: 10.3389/fpls.2026.1799303 (PMC13124708; doi:10.3389/fpls.2026.1799303)
Supplement: Supplementary file 1 [file DataSheet1.docx]

Supplementary Material

# Supplementary Data

### Supplementary Text 1. Carbon sources excluded from the metabolic analysis

Several carbon sources tested in the phenotype microarray were excluded from downstream analyses because none of the bacterial isolates reached the threshold of 50 biological units. The excluded substrates were: maltose, D-trehalose, D-cellobiose, gentiobiose, sucrose, D-turanose, stachyose, D-raffinose, ɑ-D-lactose, D-melibiose, 3-methyl-D-glucoside, D-salicin, N-acetyl-D-glucosamine, N-acetyl-D-mannosamine, N-acetyl-D-galactosamine, N-acetyl-neuraminic acid, L-fucose, L-rhamnose, D-glucose-6-phosphate, D-aspartic acid, gelatin, glycl-L-proline, pectin, 4-hydroxyphenyl acetic acid, D-lactic acid methyl ester, ɑ-hydroxybutyric acid, β-hydroxybutyric acid, ɑ-ketobutyric acid.

# Supplementary Figures and Tables

## Supplementary Tables

**Supplementary Table 1**. Sequence of primers used in the PCR amplification.

| Amplicon | Primers | References |
| --- | --- | --- |
| 16S rDNA | fD1 (5’–GAGTTTGATCCTGGCTCAG–3’);  rP1/rP2 (5’–GGYTACCTTGTTACGACTT–3’; Y=C/T) | Weisburg et al., 1991 |
| *gyrB* | Up1E (5’-CAGGAAACAGCTATGACCAYGSNGGNGGNAARTTYRA  CAGGAAACAGCTATGACC-3’);  APrU (5’-TGTAAAACGACGGCCAGTGCNGGRTCYTTYTCYTGRCA  TGTAAAACGACGGCCAGT-3’) | Yamamoto et al., 2000 |
| *rpoB* | LAPS (5-TGGCCGAGAACCAGTTCCGCGT-3);  LAPS27 (5-CGGCTTCGTCCAGCTTGTTCAG-3) | Ait Tayeb et al., 2005 |
| *rpoD* | PsEG30F (5-ATYGAAATCGCCAARCG-3)  PsEG790R (5-CGGTTGATKTCCTTGA-3) | Mulet et al., 2009 |

**Supplementary Table 2.** Inoculation scheme of representative strains, 1N3, 2N5, and 3N1, in tomato plants.

| **Inoculum (10 µL at 10^8^ CFU/ml)** | **Day 1** | **Day 2** | **Day 3** |
| --- | --- | --- | --- |
| 1N3 | 1N3 | 1N3 | 1N3 |
| 2N5 | 2N5 | 2N5 | 2N5 |
| 3N1 | 3N1 | 3N1 | 3N1 |
| 1N3+2N5 | 1N3 | 2N5 | / |
| 2N5+1N3 | 2N5 | 1N3 | / |
| 1N3+3N1 | 1N3 | 3N1 | / |
| 2N5+3N1 | 2N5 | 3N1 | / |
| 3N1+2N5 | 3N1 | 2N5 | / |
| 3N1+1N3 | 3N1 | 1N3 | / |
| 1N3+2N5+3N1 (all) | 1N3+2N5+3N1 | / | / |
| 1N3+2N5+3N1 | 1N3 | 2N5 | 3N1 |
| 1N3+3N1+2N5 | 1N3 | 3N1 | 2N5 |
| 2N5+1N3+3N1 | 2N5 | 1N3 | 3N1 |
| 2N5+3N1+1N3 | 2N5 | 3N1 | 1N3 |
| 3N1+1N3+2N5 | 3N1 | 1N3 | 2N5 |
| 3N1+2N5+1N3 | 3N1 | 2N5 | 1N3 |
| Control (water) | control | control | control |

**Supplementary Table 3.** Mean disease severity values at 24, 48, 72, 96, and 120 hours post inoculation across all bacterial treatment combinations. Values represent the mean disease severity score (S) ± standard error. A=1N3; B=2N5; C=3N1.

| **Treatment** | **24h** | **48h** | **72h** | **96h** | **120h** |
| --- | --- | --- | --- | --- | --- |
| ABC-All | 1.60 ± 0.32 | 2.73 ± 0.33 | 2.80 ± 0.29 | 2.80 ± 0.29 | 2.80 ± 0.29 |
| AC |  | 3.00 ± 0.45 | 3.00 ± 0.45 | 3.10 ± 0.40 | 3.20 ± 0.37 |
| ABC |  |  | 2.80 ± 0.34 | 3.00 ± 0.42 | 3.10 ± 0.46 |
| ACB |  |  | 2.70 ± 0.49 | 2.80 ± 0.37 | 2.80 ± 0.37 |
| AB |  | 2.40 ± 0.24 | 2.40 ± 0.24 | 2.40 ± 0.24 | 2.40 ± 0.24 |
| A | 1.83 ± 0.11 | 2.20 ± 0.13 | 2.27 ± 0.13 | 2.53 ± 0.19 | 2.63 ± 0.19 |
| C | 1.43 ± 0.23 | 2.37 ± 0.26 | 2.43 ± 0.25 | 2.47 ± 0.23 | 2.53 ± 0.23 |
| CA |  | 2.20 ± 0.20 | 2.20 ± 0.20 | 2.20 ± 0.20 | 2.40 ± 0.24 |
| CBA |  |  | 2.00 ± 0.00 | 2.20 ± 0.20 | 2.60 ± 0.40 |
| CAB |  |  | 1.80 ± 0.20 | 2.00 ± 0.16 | 2.00 ± 0.16 |
| CB |  | 1.70 ± 0.25 | 2.00 ± 0.16 | 2.40 ± 0.43 | 2.40 ± 0.43 |
| BC |  | 1.70 ± 0.37 | 2.00 ± 0.32 | 2.11 ± 0.20 | 2.22 ± 0.40 |
| B | 0.87 ± 0.14 | 1.53 ± 0.13 | 1.83 ± 0.14 | 2.00 ± 0.13 | 2.27 ± 0.19 |
| BCA |  |  | 1.90 ± 0.10 | 2.00 ± 0.16 | 2.00 ± 0.16 |
| BA |  | 1.40 ± 0.19 | 1.70 ± 0.20 | 2.10 ± 0.19 | 2.90 ± 0.33 |
| BAC |  |  | 2.00 ± 0.00 | 2.20 ± 0.20 | 2.60 ± 0.40 |

**Supplementary Table 4.** Nutritional compatibility of the three *Pseudomonas* species.

| **Strain** | **N. utilized carbon sources** | **N. shared carbon sources** | **NOI** |
| --- | --- | --- | --- |
| **1N3** | 39 | 37 | 0.948718 |
| **2N5** | 40 |  | 0.925 |
| **3N1** | 42 | 39 | 0.928571 |
| **2N5** | 40 |  | 0.975 |
| **3N1** | 42 | 39 | 0.928571 |
| **1N3** | 39 |  | 1 |

## Supplementary Figures


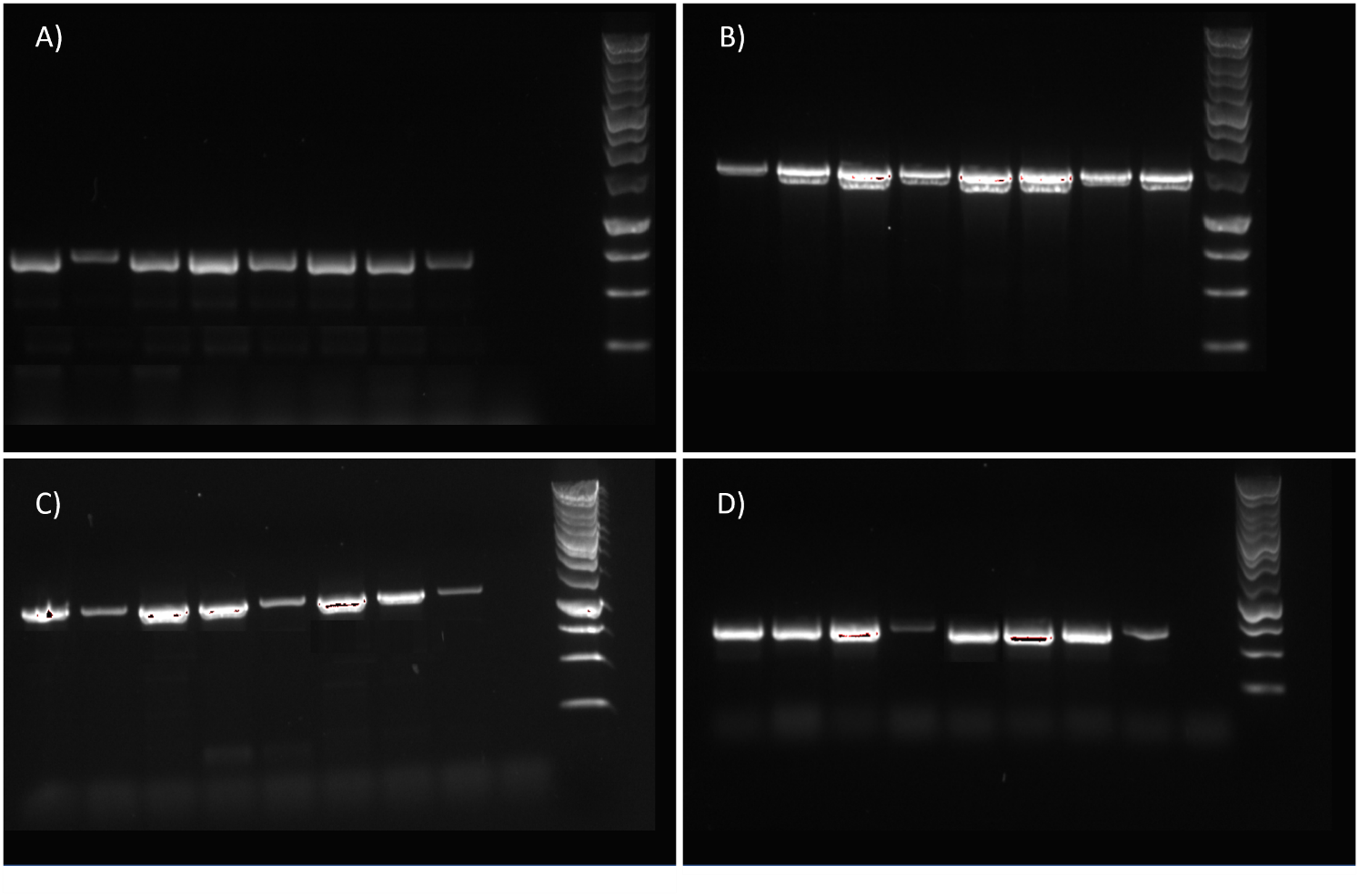


**Supplementary Figure 1.** Gel electrophoresis images of A) *rpoD*, B) 16S rDNA, C) *rpoB*, and D) *gyrB* gene amplicons. From left to right:1N2, 1N3, 1N4, 2N9, 2N5, 2N8, 3N1, and 3N3, 1Kb DNA Ladder (Promega Corporation).

**
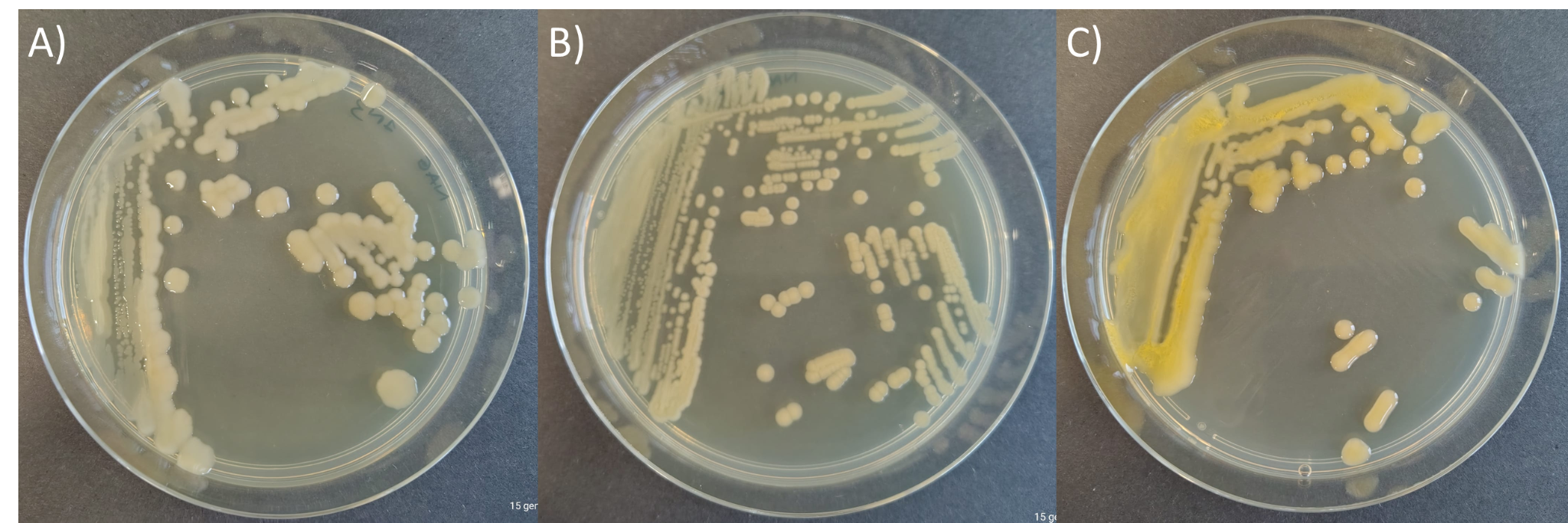
**

**Supplementary Figure 2.** Bacterial isolate morphologies on NGA medium. (A) *Pseudomonas alliivorans* 1N3; (B) *P. asturiensis* 2N5; (C) *P. viridiflava* 3N1.

*
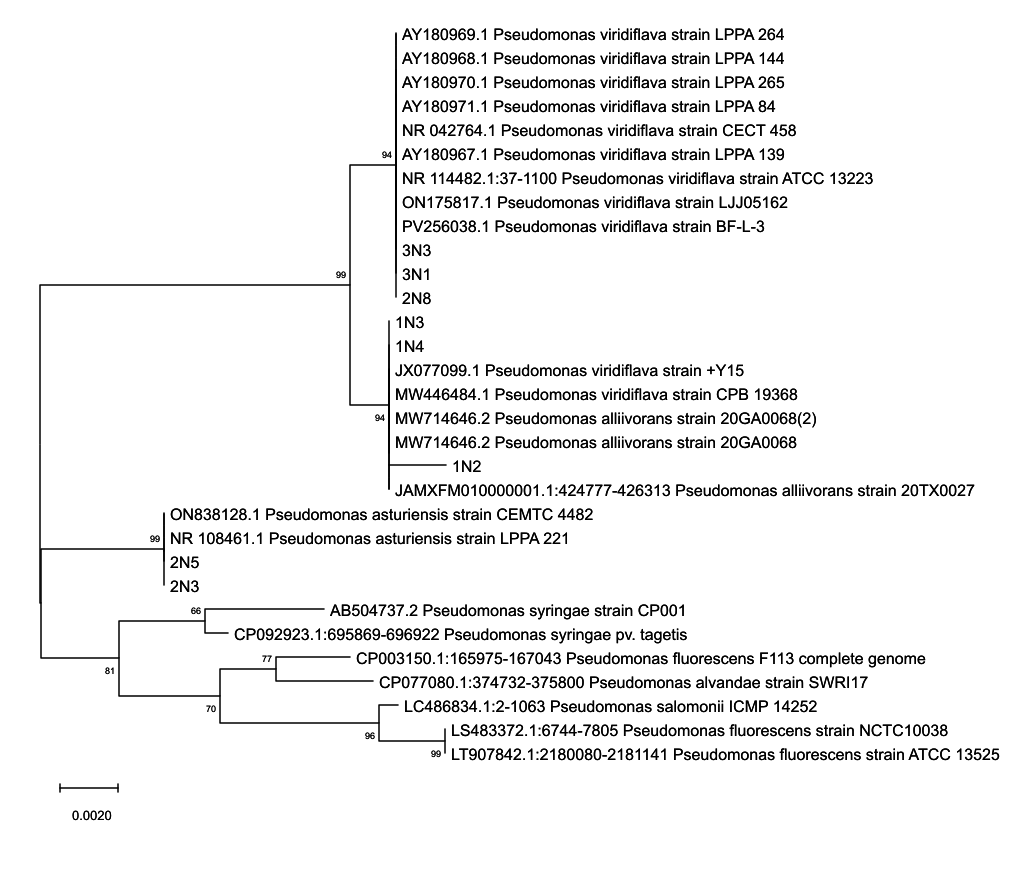
*

**Supplementary Figure 3**. Phylogenetic tree obtained using 16S rDNA sequences (1025 bp). The percentage of replicate trees in which the associated taxa clustered together in the bootstrap test (1,000 replicates) is shown next to the branches. Trees are drawn to scale, with branch lengths expressed in the same units as those used to compute evolutionary distances. Distances are reported in units of base substitutions per site.

*
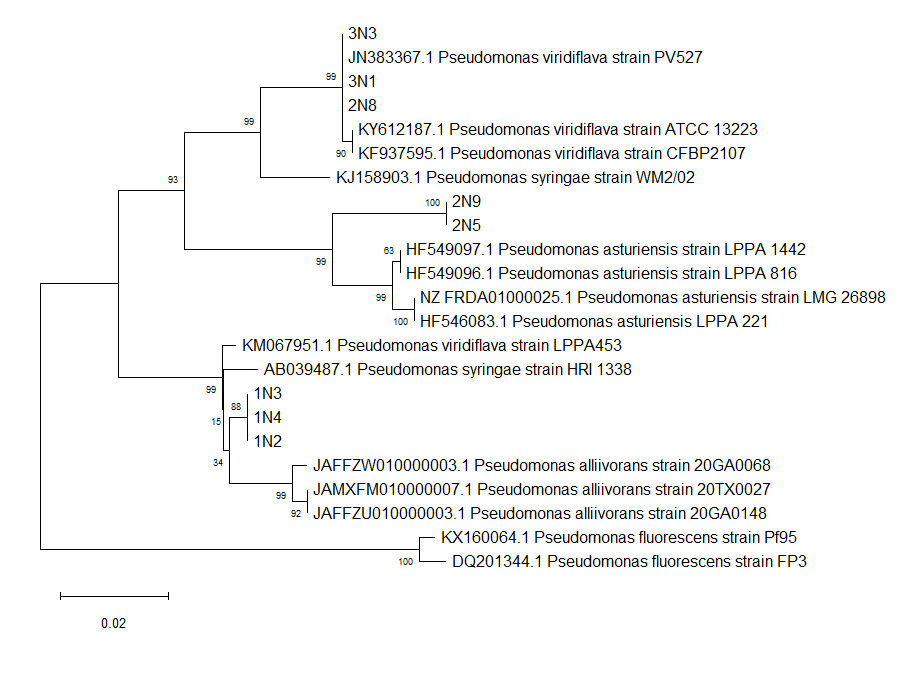
***Supplementary Figure 4**. Phylogenetic tree obtained using *gyrB* sequences (550 bp). The percentage of replicate trees in which the associated taxa clustered together in the bootstrap test (1,000 replicates) is shown next to the branches. Trees are drawn to scale, with branch lengths expressed in the same units as those used to compute evolutionary distances. Distances are reported in units of base substitutions per site.

*
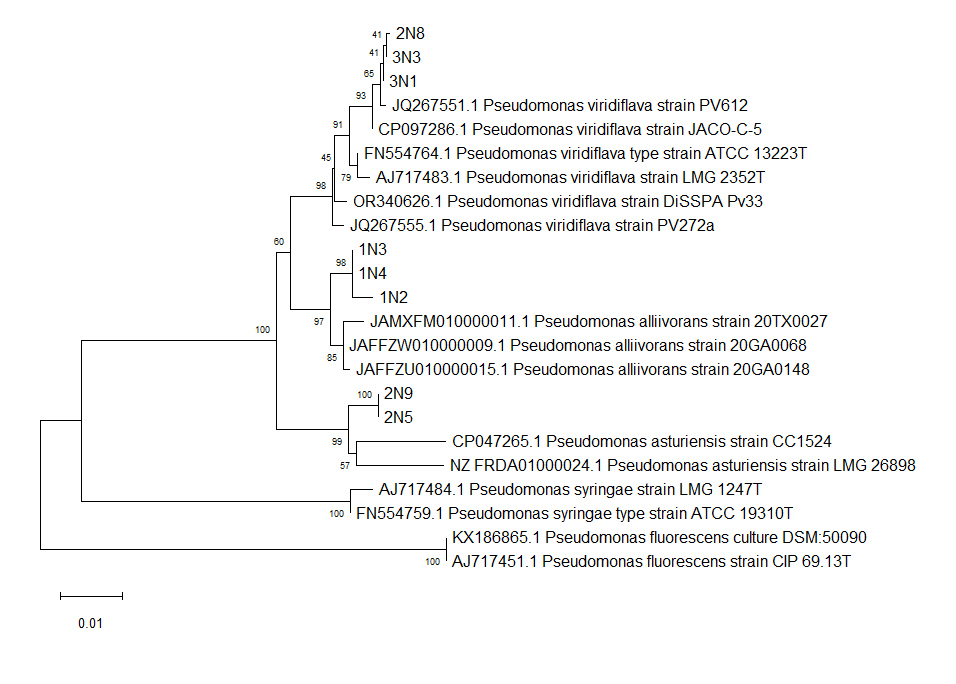
***Supplementary Figure 5**. Phylogenetic tree obtained using *rpoB* sequences (474 bp). The percentage of replicate trees in which the associated taxa clustered together in the bootstrap test (1,000 replicates) is shown next to the branches. Trees are drawn to scale, with branch lengths expressed in the same units as those used to compute evolutionary distances. Distances are reported in units of base substitutions per site.

*
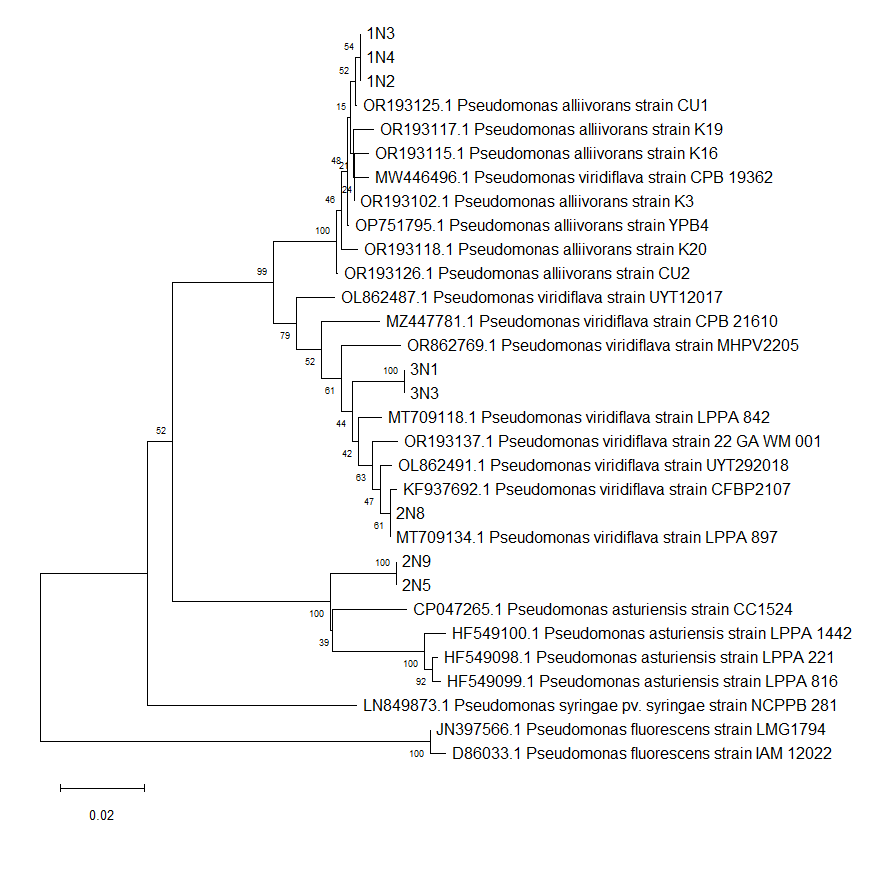
***Supplementary Figure 6**. Phylogenetic tree obtained using *rpoD* sequences (570 bp). The percentage of replicate trees in which the associated taxa clustered together in the bootstrap test (1,000 replicates) is shown next to the branches. Trees are drawn to scale, with branch lengths expressed in the same units as those used to compute evolutionary distances. Distances are reported in units of base substitutions per site.


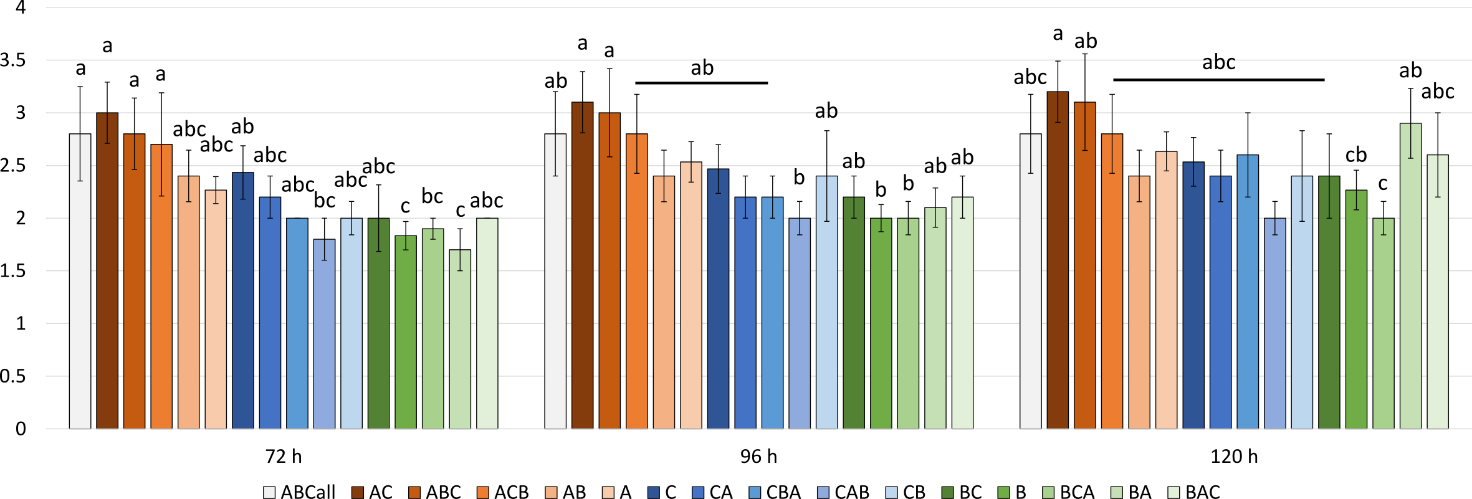


**Supplementary Figure 7**. Mean disease severity values at 72, 96, and 120 hours post inoculation across all bacterial treatment combinations. Bars represent the mean disease severity score (S), while error bars indicate the standard error. Statistical differences were evaluated using a Kruskal–Wallis test followed by Dunn’s post hoc test. Different letters above error bars indicate statistically significant differences among conditions within the same time point (p < 0.05). A=1N3; B=2N5; C=3N1.
